# Mitochondrial Dysfunctions in Human Primary Coenzyme Q_10_ Deficiencies

**DOI:** 10.3390/biom16020302

**Published:** 2026-02-14

**Authors:** Fanny Fontaine, Romain Pénicaud, Stéphane Allouche

**Affiliations:** 1 Department of Clinical Biochemistry, CHU Caen Normandie, CS 30001, 14033 Caen Cedex, France; fontaine-fa@chu-caen.fr (F.F.); penicaud-r@chu-caen.fr (R.P.); 2 UMRS 1237—Physiopathologie et Imagerie des Troubles Neurologiques (PHIND), Université de Caen Basse-Normandie, 14000 Caen, France

**Keywords:** coenzyme Q_10_, primary coenzyme Q_10_ deficiency, mitochondrial disorders, oxidative phosphorylation, oxidative stress, mitophagy, metabolism

## Abstract

Coenzyme Q_10_ (CoQ_10_) is an essential lipid-soluble molecule that plays a central role in mitochondrial energy production as a mobile electron carrier. In addition to its bioenergetic function, CoQ_10_ participates in antioxidant defense, redox homeostasis, lipid and nucleotide metabolism, and mitochondrial quality control. Primary CoQ_10_ deficiencies are rare inherited mitochondrial disorders caused by pathogenic variants in nuclear genes involved in CoQ_10_ biosynthesis. These defects lead to reduced CoQ_10_ levels and impaired mitochondrial functions. Clinically, primary CoQ_10_ deficiencies display remarkable phenotypic heterogeneity, ranging from isolated organ involvement, notably renal or cerebellar disease, to severe multisystemic disorders affecting the nervous system, skeletal muscle, heart, and other organs. Disease onset spans from the antenatal period to adulthood, and clinical severity varies widely, even among patients carrying variants in the same gene. This diversity cannot be fully explained by defective ATP production alone. Growing evidence indicates that disruption of non-bioenergetic functions of CoQ_10_, including oxidative stress regulation and CoQ-dependent metabolic pathways, contributes significantly to disease pathophysiology and tissue vulnerability. In this review, we summarize current knowledge on CoQ_10_ biology, biosynthesis, and the clinical spectrum of primary CoQ_10_ deficiencies, and we discuss emerging mechanisms linking CoQ_10_ depletion to mitochondrial dysfunctions and human diseases.

## 1. Introduction

CoQ_10_, also known as ubiquinone/ubiquinol, is a ubiquitous and essential lipid-soluble molecule that plays a central role in cellular metabolism. Best known for its function as a mobile electron carrier within the mitochondrial respiratory chain (MRC), CoQ_10_ is indispensable for oxidative phosphorylation and ATP production. Beyond its bioenergetic role, CoQ_10_ participates in a wide range of cellular processes, including antioxidant defense, regulation of redox homeostasis, lipid metabolism, and the activity of several mitochondrial and extra-mitochondrial enzymes. Given its multifaceted functions and ubiquitous distribution, it is not surprising that disturbances in CoQ_10_ homeostasis have profound consequences for cellular functions and physiology.

Primary CoQ_10_ deficiency is a rare group of inherited mitochondrial disorders caused by pathogenic variants in nuclear genes encoding proteins involved in CoQ_10_ biosynthesis. To date, mutations in 11 genes of this pathway have been identified in humans, leading to reduced CoQ_10_ levels in tissues and impaired mitochondrial functions. Clinically, primary CoQ_10_ deficiency is remarkable for its extreme phenotypic heterogeneity. Patients may present with isolated organ involvement, notably the kidney or the cerebellum, or with severe, multisystemic disease affecting the central and peripheral nervous systems, skeletal muscle, heart, and other organs. Age at onset ranges from the antenatal or neonatal period to adulthood, and disease severity varies from rapidly fatal forms to slowly progressive or relatively stable phenotypes.

This clinical variability contrasts with the shared biochemical defect underlying these disorders and raises fundamental questions regarding disease mechanisms. While impaired mitochondrial ATP production is a key consequence of CoQ_10_ deficiency, it alone cannot fully explain the tissue specificity and phenotypic diversity observed in affected individuals. Increasing evidence suggests that disruption of non-bioenergetic functions of CoQ_10_, including its roles in redox signaling, lipid and nucleotide metabolism, mitochondrial quality control, and oxidative stress regulation, also contributes substantially to disease pathophysiology. Moreover, differences in residual CoQ_10_ levels, tissue-specific metabolic demands, and the stability of the CoQ biosynthetic complex likely modulate disease expression.

In this review, we provide an updated and comprehensive overview of mitochondrial dysfunctions associated with primary CoQ_10_ deficiencies in humans. We first summarize the biochemical properties, cellular localization, and biosynthesis of CoQ_10_, with particular emphasis on the organization of the CoQ biosynthetic machinery. We then review the clinical spectrum of primary CoQ_10_ deficiencies, highlighting genotype–phenotype correlations and organ-specific manifestations. Finally, we discuss current pathophysiological hypotheses linking CoQ_10_ deficiency to mitochondrial and cellular dysfunctions, with the aim of clarifying the mechanisms underlying tissue vulnerability and phenotypic variability in these disorders.

## 2. About Coenzyme Q_10_

### 2.1. Structure

**Figure 1:**
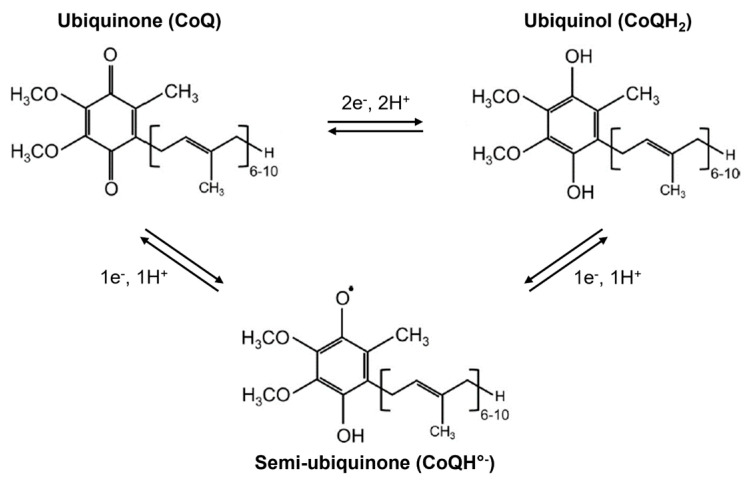
Redox states of CoQ. Ubiquinone (CoQ) represents the fully oxidized form, Ubiquinol (CoQH_2_) the fully reduced form, and semiquinone (CoQH°^−^) the intermediate radical form. Depending on the species, the side chains contain between 6 and 10 isoprene units.

CoQ_10_ was discovered in 1955 when Festenstein and colleagues isolated and described a “substance SA” from lipid fractions of various animal tissues (rat, pig, horse) [1]. This compound was characterized as relatively non-polar, possibly a hydrocarbon, steroid, or fat-soluble vitamin. Infrared spectral analysis suggested the presence of a chromophore with conjugated double bonds linked to one or two ketone groups, alongside one or more isopropyl side chains. Two years later, Crane et al. extracted a “quinone compound Q-275” (absorbing at 275 nm in ethanol) from beef heart mitochondria and demonstrated its role as a coenzyme in the mitochondrial respiratory chain through reversible redox cycling (Figure 1) [2]. They further identified this compound and four homologues in yeasts and bacteria, collectively termed coenzyme Q or ubiquinones [3].

The molecular structure of CoQ was definitively characterized in 1958 by Wolf and colleagues [4]. Ubiquinones are amphipathic organic molecules composed of a polar headgroup and a hydrophobic isoprenoid side chain. The general structure features a 2-methyl-5,6-dimethoxy-1,4-benzoquinone ring substituted at position 3 by an isoprenoid chain, the length of which varies between species (Figure 1). This side chain typically contains 6 to 10 isoprene units, a feature that underlies the nomenclature CoQ_n_, where *n* denotes the number of isoprenoid repeats. In humans, CoQ_10_ is the predominant active form. By contrast, rodents and *Caenorhabditis elegans* primarily produce CoQ_9_ [5], Enterobacteria synthesize CoQ_8_ [6], and *Saccharomyces cerevisiae* produces CoQ_6_ [7]. The benzoquinone ring constitutes the redox-active center of CoQ, capable of existing in three principal oxidation states: fully oxidized ubiquinone (CoQ), partially reduced semiquinone radical (CoQH°^−^), and fully reduced ubiquinol (CoQH_2_) bearing two hydroxyl groups (Figure 1). This reversible redox cycling, involving one- or two-electron transfers without structural alteration of the molecule, underpins CoQ’s roles as a coenzyme, mobile electron carrier in the MRC, and antioxidant [8].

### 2.2. Localization

The total pool of CoQ_10_ in humans is estimated to range between 0.5 and 1.5 g, distributed predominantly between a large tissue reservoir and a smaller circulating blood pool. CoQ is ubiquitous and is present in virtually all tissues and cells. In humans, the highest concentrations are found in organs with high energy demands, such as the heart, liver, skeletal muscles, neurons and kidneys [5]. The heart, in particular, is exceptionally rich in CoQ_10_, where it plays a vital role in ATP (adenosine triphosphate) production necessary for myocardial contraction through oxidative phosphorylation. CoQ_10_ levels also vary considerably among cellular compartments, with the highest concentrations localized in the inner mitochondrial membrane, the site of the electron transport chain (ETC). It is also found in lysosomes, the Golgi apparatus, and the endoplasmic reticulum, where it acts as an antioxidant and an enzymatic cofactor, beyond bioenergetics [9]. In the bloodstream, CoQ_10_ circulates mainly bound to lipoproteins, with approximately 76% found in the plasma fraction and 24% within blood cells. Within plasma from fasting subjects, about 58% of CoQ_10_ associates with low-density lipoproteins (LDLs), 26% with high-density lipoproteins (HDLs), and 16% with other lipoprotein particles [10].

Within tissues and cells, CoQ_10_ is associated with plasma membranes, where it acts as a potent antioxidant, protecting lipids and proteins from oxidative damage, which is especially important in tissues exposed to high metabolic rates and reactive oxygen species (ROS, see Section 5.2.3). However, its precise intramembrane localization is not fully elucidated and seems to vary depending on its redox state. Due to its amphipathic nature, CoQ_10_ likely adopts a configuration analogous to phospholipids, with the polar benzoquinone headgroup oriented toward the membrane surface and the hydrophobic isoprenoid tail embedded within the membrane core [11,12]. Molecular dynamics simulations have shown that ubiquinol tends to localize near the membrane surface, stabilized by hydrogen bonding with phosphoryl groups of glycerophospholipids, whereas the ubiquinone resides more centrally within the lipid bilayer [13]. CoQ_10_ exhibits rapid lateral and transbilayer mobility within membranes, allowing it to efficiently shuttle electrons between enzymatic complexes of the respiratory chain, necessary for ATP synthesis [14].

It is also important to note that CoQ_10_ levels are dynamic and influenced by several factors. Aging is associated with a gradual decline in CoQ_10_ tissue concentrations, potentially contributing to reduced mitochondrial efficiency and increased oxidative stress [15]. Dietary intake, CoQ_10_ supplementation, and pathological states such as mitochondrial diseases, cardiovascular disorders, and neurodegenerative conditions can significantly affect CoQ_10_ levels, highlighting its clinical relevance [16].

### 2.3. Pharmacokinetics

Although most CoQ_10_ in the body is synthesized endogenously, a small amount is obtained from the diet. Typical dietary intake is low, around 3–5 mg per day, mainly from meat, poultry, fatty fish, and some vegetables and fruits [17]. Dietary CoQ_10_ can contribute up to 25% of the plasma CoQ_10_ pool [18]. CoQ_10_ has poor intestinal absorption due to its large molecular weight and hydrophobic nature, with bioavailability estimated at only 2–3%. Absorption is enhanced when CoQ_10_ is ingested with lipids, following a process similar to fat-soluble vitamins. In the small intestine, bile and pancreatic secretions emulsify CoQ_10_ into micelles, which are passively absorbed by enterocytes. Inside these cells, CoQ_10_ is reduced to ubiquinol and incorporated into chylomicrons that enter the lymphatic system and subsequently the bloodstream. Chylomicrons are metabolized by lipoprotein lipase, and CoQ_10_ is transferred to LDL and very-low-density lipoprotein (VLDL) particles for systemic distribution [19]. Peak plasma concentrations are typically reached 6 to 8 h post-ingestion, with a secondary peak around 24 h due to enterohepatic recycling [20]. However, CoQ_10_ plasma levels increase nonlinearly with dosage, and prolonged supplementation (2–4 weeks) leads to steady-state plasma concentrations without tissue accumulation [21]. CoQ_10_ exhibits a plasma half-life of approximately 33 h in humans [22], and elimination occurs mainly via the biliary-fecal route (90–95%) either unchanged or as glucuronide conjugates, while a smaller fraction (5–10%) is excreted renally as phosphorylated metabolites [23].

The circulating plasma pool contributes minimally to the tissue CoQ_10_ pool under normal physiological conditions. Tissue uptake of exogenous CoQ_10_ is limited, with significant distribution mainly observed in the liver, spleen, and circulating white blood cells, while energy-demanding tissues such as the heart, brain, and skeletal muscles show poor absorption unless high doses are administered [24,25]. This explains the challenges in achieving adequate tissue levels through oral supplementation. Endogenous synthesis remains the primary source of CoQ_10_ in tissues, and exogenous supplementation does not downregulate this production. However, in specific pathological conditions such as primary CoQ_10_ deficiency (due to biosynthetic gene mutations) or increased demand (due to imbalanced redox status), oral supplementation (especially with the more bioavailable reduced form, ubiquinol) can partially restore tissue levels and improve clinical outcomes [26].

## 3. Biosynthesis of Coenzyme Q_10_

In mammalian cells, tissue CoQ_10_ is mainly provided by the biosynthetic pathway. CoQ_10_ biosynthesis is a multi-step and evolutionarily conserved process involving biochemical reactions that take place in the cytosol and the mitochondrial matrix, although evidence suggests that limited synthesis may also take place in other subcellular compartments [27]. In humans, CoQ_10_ biosynthesis involves at least ten dedicated enzymes, many of which (COQ3, COQ4, COQ5, COQ6, COQ7, COQ8, COQ9) are assembled into a large multiprotein structure known as complex Q, or the Q synthome (see Section 3.5) [28]. This assembly exemplifies a metabolon: a dynamic and transient complex that enables sequential enzymatic reactions through substrate channeling, thereby improving metabolic efficiency and minimizing the release of potentially harmful intermediates [29]. Those enzymes, which are encoded by nuclear genes, contain a mitochondrial targeting sequence that enables their importation into the mitochondria via translocases of the outer and the inner membranes [30].

**Figure 2:**
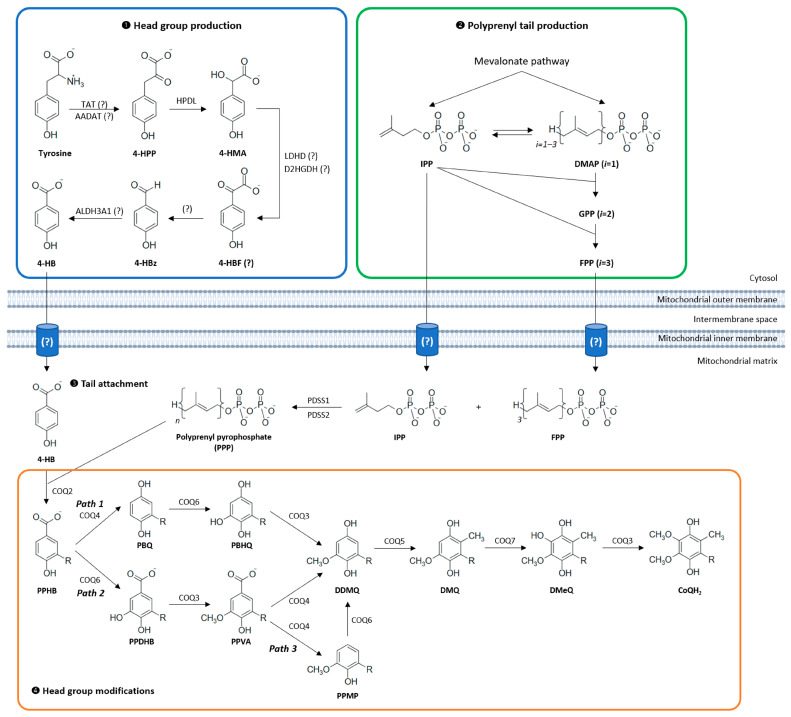
Coenzyme Q_10_ biosynthetic pathway in humans. The (?) indicates steps that are catalyzed by unknown enzyme(s) or unknown synthetic intermediates, thus resulting in an uncertainty in the order of reactions. Intermediates in the pathway include 4-HPP (4-hydroxyphenylpyruvate), 4-HMA (4-hydroxymandelate), 4-HBF (4-hydroxybenzoylformate), 4-HBz (4-hydroxybenzaldehyde), 4-HB (4-hydroxybenzoate), DMAP (dimethylallylpyrophosphate), IPP (isopentenyl pyrophosphate), GPP (geranylpyrophosphate), FPP (farnesylpyrophosphate), PPHB (3-polyprenyl-4-hydroxy-benzoate), PBQ (3-polyprenyl-1,4-benzohydroquinone), PBHQ (3-polyprenyl-5-hydroxy-1,4-benzohydroquinone), PPDHB (3-polyprenyl-4,5-dyhydroxybenzoate), PPVA (3-polyprenylvanillic acid), PPMP (2-polyprenyl-6-methoxyphenol), DDMQ (Demethoxy-demethylCoQ_10_, 3-polyprenyl-5-methoxy-1,4-benzohydroquinone), DMQ (DemethoxyCoQ_10_, 2-methyl-3-polyprenyl-5-methoxy-1,4-benzohydroquinone), DMeQ (DemethylCoQ_10_, 2-methyl-3-polyprenyl-5-methoxy-6-hydroxy-1,4-benzohydroquinone). Enzymes include TAT (tyrosine aminotransferase), AADAT (alpha-aminoadipate aminotransferase), HPDL (hydroxyphenylpyruvate dioxygenase-like protein), LDHD (lactate dehydrogenase D), D2HGDH (D-2-hydroxyglutarate dehydrogenase), ALDH3A1 (aldehyde dehydrogenase 3 Family Member 1).

This biosynthetic pathway can be broadly divided into four stages: (1) synthesis of the aromatic head group, (2) production of the isoprenoid tail, (3) attachment of the head group to the tail, and (4) a series of modifications to the ring structure (Figure 2) [31]. Much of our understanding of this pathway derives from studies in model organisms such as *Escherichia coli* and *Saccharomyces cerevisiae*, using CoQ-deficient mutants [32,33]. Although the enzymatic functions and characteristics of human proteins implicated in CoQ_10_ biosynthesis are poorly understood, their functions have been indirectly verified by the restoration of CoQ production after expressing the human genes in their corresponding yeast *coq* null mutants and by studying patients’ fibroblasts with pathogenic variants in *COQ* genes. In yeast, most of the CoQ biosynthetic proteins are referred to as “CoqX”, derived from the corresponding *coqX* genes, whereas in humans, the nomenclature follows “COQX” for proteins and *COQX* for genes. Complete disruption of CoQ biosynthesis is embryonically lethal in most animal models, underlining its vital role in cellular metabolism. Despite significant advances, many aspects of CoQ_10_ biosynthesis, transport, and regulation remain incompletely understood, posing challenges for the effective treatment of primary CoQ_10_ deficiencies in humans.

### 3.1. Head Group Production

The biosynthesis of CoQ_10_ universally relies on the conserved head group precursor 4-hydroxybenzoate (4-HB), which is derived from tyrosine in eukaryotic cells. In humans, the presence of phenylalanine hydroxylase (PAH), which converts phenylalanine into tyrosine, enables the use of both amino acids as potential precursors for 4-HB production [34]. While the overall pathway from tyrosine to 4-HB is conserved, several steps remain incompletely understood.

In human cells, tyrosine aminotransferase (TAT) and alpha-aminoadipate aminotransferase (AADAT) are considered plausible candidates for catalyzing the initial transamination of tyrosine to 4-hydroxyphenylpyruvate (4-HPP). Recent metabolic tracing experiments using isotopically labeled oxygen identified 4-hydroxymandelate (4-HMA) as a novel intermediate, formed from 4-HPP via the hydroxyphenylpyruvate dioxygenase-like protein (HPDL). The conversion of 4-HMA to 4-HBz is proposed to proceed through 4-hydroxybenzoylformate (4-HBF), potentially catalyzed by lactate dehydrogenase D (LDHD) or D-2-hydroxyglutarate dehydrogenase (D2HGDH) [35]. The final oxidation step, from 4-HBz to 4-HB, may be mediated by aldehyde dehydrogenase 3 Family Member A1 (ALDH3A1), which has been shown to catalyze this reaction in vitro. However, its physiological role in CoQ_10_ biosynthesis in mammalian cells has yet to be firmly established [36].

Beyond 4-HB, several natural aromatic compounds can serve as alternative precursors for CoQ biosynthesis, such as resveratrol, p-coumaric acid and kaempferol. These dietary polyphenols are thought to be metabolized into 4-HB, thereby offering an alternative source of the aromatic ring in conditions of impaired endogenous 4-HB synthesis [37,38].

### 3.2. Isoprenoid Tail Production

The mevalonate pathway comprises a series of enzymatic reactions that convert acetyl-CoA into farnesyl pyrophosphate (FPP) [39]. The long isoprenoid tail of CoQ_10_ originates from polyprenyl pyrophosphate (PPP), which results from the condensation of isopentenyl pyrophosphate (IPP) with FPP [40]. This reaction is catalyzed by a heterotetramer composed of the protein subunits *PDSS1* and *PDSS2* (decaprenyl-diphosphate synthase subunits 1 and 2) [41]. The mechanisms by which both 4-HB and isoprenoid precursors are transported from the cytosol into the mitochondrial matrix remain unknown.

### 3.3. Attachment of the Isoprenoid Tail to the Head Group Precursor

The subsequent step in CoQ_10_ biosynthesis is the conjugation of the decaprenylpyrophosphate chain to 4-HB, resulting in the formation of 3-polyprenyl-4-hydroxybenzoate (PPHB), the first prenylated intermediate in the pathway. This reaction is catalyzed by the parahydroxybenzoate-polyprenyltransferase *COQ2*, an integral protein of the inner mitochondrial membrane (IMM) whose active site is oriented toward the mitochondrial matrix [42,43,44]. The availability of endogenous 4-HB directly influences the rate of this reaction, making it a key rate-limiting step in CoQ production [45]. COQ2 exhibits broad substrate specificity and can prenylate alternative exogenous aromatic substrates such as 2,4-dihydroxybenzoic acid (2,4-diHB), 3,4-dihydroxybenzoic acid (3,4-diHB), and vanillic acid. These hydroxylated or methoxylated 4-HB analogs may bypass specific enzymatic blocks within the CoQ biosynthetic pathway [46].

### 3.4. Head Group Modifications

The final phase of CoQ_10_ biosynthesis entails a sequence of modifications of the PPHB head, including decarboxylation, hydroxylation at three positions, and three methylation reactions, that are mediated by COQ proteins. In both bacteria and yeast, the methyl and hydroxyl groups added during these modifications are derived from S-adenosyl methionine (SAM) and molecular oxygen (O_2_), respectively, a mechanism that is also presumed to operate in mammals [47,48].

The proposed sequence of enzymatic modifications is inferred from the identification of CoQ intermediates in bacterial and yeast mutants and generally aligns with the chemical principles of electrophilic aromatic substitution. However, the precise order in which these modifications occur remains under debate. Classical models based on yeast data suggest that the first modification is a C5 hydroxylation catalyzed by COQ6, followed by a methylation step mediated by COQ3, and subsequently the decarboxylation and hydroxylation at C1 (Figure 2—path 2) [33,49]. Yet, this sequence may be more flexible than previously thought: in both yeast and human cells deficient in COQ6, 3-polyprenyl-1,4-benzohydroquinone (PBQ) accumulates, indicating that C1 decarboxylation and hydroxylation can, in some contexts, occur prior to C5 modifications (Figure 2—path 1). This flexibility was further supported by recent findings implicating COQ4 in the decarboxylation/hydroxylation step [50].

COQ4, originally thought to act only as a structural chaperone within the CoQ biosynthetic complex, has been shown to have a direct enzymatic role in CoQ_10_ synthesis [51,52]. Studies in HEK293 cells demonstrate that COQ4 catalyzes the oxidative decarboxylation of prenylated CoQ_10_ precursors: the loss of COQ4 blocks CoQ_10_ production and causes strong accumulation of PPHB, indicating that COQ4 converts PPHB to PBQ in a single decarboxylation step. The preferential accumulation of PPHB in COQ4-deficient cells, together with the PBQ accumulation observed in COQ6-deficient cells, positions COQ4 early in the sequence of head-group modifications (Figure 2—Path 1), preceding COQ6-mediated hydroxylation, although alternative pathways consistent with yeast models (Figure 2—Path 2) remain possible [50]. Additional evidence shows that COQ4 can process prenylated substrates with a C5 methoxy group and that COQ6 may have both C5 and C1 hydroxylation activity (Figure 2—Path 3) [28,53]. Overall, the data suggest that COQ4 has a core catalytic function and that the eukaryotic CoQ pathway may exhibit flexibility in the ordering of enzymatic steps.

The penultimate step in the CoQ biosynthetic pathway is the hydroxylation at the C6 position, catalyzed by the COQ7 enzyme [54,55]. In human fibroblasts with COQ7-associated CoQ_10_ deficiencies, accumulation of demethoxyCoQ10 (DMQ) was observed, and treatment with 2,4-dihydroxybenzoate (2,4-diHB, a 4-HB analog already hydroxylated at C6) improved CoQ_10_ levels. The efficacy of this treatment depends on the specific COQ7 mutations present in patients. The success of such bypass therapy likely requires a stable presence of other COQ proteins and their ability to assemble into the Q complex [56,57].

The hydroxyl groups introduced at positions C5 and C6 are subsequently methylated by the COQ3 enzyme [58,59]. To date, no mutations in the human *COQ3* gene have been identified as a cause of primary CoQ_10_ deficiency. Methylation at the C2 position is catalyzed by the COQ5 enzyme, whose activity depends on the structural integrity and stability of the CoQ synthome [60].

### 3.5. The Complex Q

Building on findings in yeast, research suggests that human cells also utilize a “complex Q” for CoQ_10_ biosynthesis. This multi-protein assembly consists of enzymes, chaperone proteins, CoQ_10_ itself, and its biosynthetic intermediates. While the core complex includes COQ3 through COQ9, proteins such as COQ1, COQ2, and COQ10 operate independently [61]. In a study using human 143B cells, transient knockdown of *PDSS1*, *PDSS2*, and several *COQ* genes led to altered expression levels of other COQ proteins, with both decreases and compensatory increases observed. Notably, PDSS1, PDSS2, and COQ3 appear essential for maintaining the stability of the overall complex. Interestingly, COQ5 protein levels were reduced following *PDSS1*, *PDSS2*, and *COQ9* knockdown. These results also revealed a potential functional interplay between COQ3 and COQ6, as the loss of one protein destabilized the other. Moreover, the mechanisms underlying COQ5 and COQ6 upregulation after *COQ4* knockdown, as well as increased COQ4 levels following *COQ7* knockdown, remain unclear. These effects may reflect compensatory regulatory mechanisms aimed at preserving CoQ_10_ biosynthesis under conditions of partial deficiency. The observation that COQ5 expression is particularly sensitive to moderate and transient COQ9 loss underscores the underappreciated role of COQ9 in stabilizing the complex [62]. This is further supported by other studies reporting reduced COQ5 levels in fibroblasts from patients with *COQ9* deficiency [63]. Altogether, the organization, stability, and protein–protein interactions within complex Q appear to be highly dynamic and context-dependent, likely varying across different cellular models and physiological and pathological conditions.

### 3.6. Other Proteins Implicated in Coenzyme Q_10_ Biosynthesis

Other proteins were suggested to participate in the CoQ_10_ biosynthetic pathway, including ADCK (aarF domain-containing kinase) proteins, which share structural features with kinase-like proteins. Although COQ8A and COQ8B (formerly known as ADCK3 and ADCK4) are required for CoQ_10_ production, their precise roles remain unresolved. In humans, COQ8A has no kinase activity in vitro but instead exhibits ATPase activity when bound to cardiolipins, suggesting that ATP hydrolysis helps to recruit CoQ_10_ precursors and assemble the CoQ_10_ biosynthetic machinery, functioning in a chaperone-like manner [64]. Likewise, COQ8B shows ATPase activity and was hypothesized to act as a COQ3 kinase [28]. Both *COQ8A* and *COQ8B* genes have been associated with primary CoQ_10_ deficiency with completely different phenotypes. *COQ8A* pathogenic variations cause cerebellar ataxia and encephalopathy, whereas *COQ8B* mutations mainly cause steroid-resistant nephrotic syndrome (see Section 4). *ADCK2* haploinsufficiency was shown to cause an adult-onset myopathy with CoQ_10_ deficiency and defects in mitochondrial fatty acid β-oxidation, revealing another clinical phenotype for *ADCK* pathogenic variations [65].

In humans, COQ9 has been shown to physically interact with COQ7 and is essential for the hydroxylation step catalyzed by this protein [66]. COQ9 contains a hydrophobic lipid-binding pocket that can accommodate various lipids, including CoQ intermediates, suggesting a role in the transport or presentation of these intermediates to other COQ enzymes. Cryo-EM studies have revealed that COQ9 and COQ7 interact through a well-defined interface stabilized by hydrogen bonds, hydrophobic contacts, and an intermolecular salt bridge [67]. Functional interdependence between the two proteins is further supported by the accumulation of DMQ (the substrate of COQ7) in fibroblasts deficient for either COQ7 or COQ9. Both deficiencies can be partially rescued by treatment with 2,4-diHB, a hydroxylated analog of 4-HB that bypasses the COQ7-catalyzed hydroxylation step [57,68].

While in yeast the ortholog of the two isoforms COQ10A and COQ10B was suggested to act as a chaperone-like protein by allowing binding of CoQ intermediates, no disease-causing mutations have been reported for those genes in humans [69].

### 3.7. Extra-Mitochondrial Coenzyme Q_10_ Biosynthesis

Cell fractionation studies have demonstrated that while CoQ_10_ is primarily concentrated within the mitochondria, it is also distributed, albeit to a lesser extent, to extra-mitochondrial compartments, notably the plasma membrane, where it functions as an antioxidant [70,71]. This extra-mitochondrial presence has been hypothesized to result from an alternative biosynthetic pathway. For instance, Mugoni et al. identified the *ubiad1* gene in zebrafish, showing that its deletion causes oxidative stress-induced cardiovascular lesions. In humans, the Golgi-localized prenyltransferase UBIAD1 is thought to facilitate this alternative synthesis [72]. However, since this pathway remains unconfirmed in other experimental models, its contribution may be uniquely restricted to cardiac tissue.

### 3.8. Intracellular Coenzyme Q_10_ Transport

Following its synthesis within the mitochondria, CoQ_10_ is redistributed to various intracellular compartments. A recent study by Deshwal et al. identified STARD7, a protein localized in both the mitochondrial intermembrane space and the cytosol, as a key factor of this transport process [73]. The mitochondrial form of STARD7 is indispensable for CoQ_10_ biosynthesis and respiratory cell growth. In contrast, its cytosolic counterpart facilitates the trafficking of CoQ_10_ to the plasma membrane, where it serves as a robust antioxidant, protecting membrane phospholipids from peroxidation and inhibiting ferroptotic cell death (see Section Antioxidant Systems). However, we cannot rule out the possibility that the extra-mitochondrial localization of CoQ_10_ results from lipid exchange between the mitochondria and other intracellular compartments. Extensive evidence has demonstrated that mitochondria and the endoplasmic reticulum (ER) maintain close contact through structures known as mitochondria-associated membranes (MAMs), which facilitate lipid exchange between these organelles [74]. Lipids within the ER can then be transported to the plasma membrane (PM) either via the secretory pathway or directly through ER-PM contact sites. Current data remain insufficient to determine if CoQ_10_ levels across these compartments exist in dynamic equilibrium or if independent regulatory mechanisms allow for the selective control of CoQ_10_ in one compartment without influencing others.

## 4. Clinical Features and Phenotypes Associated with Primary Coenzyme Q_10_ Deficiency in Humans

**Figure 3:**
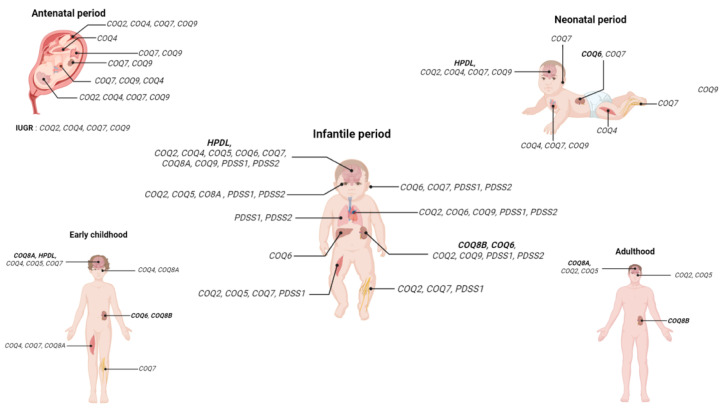
The organs affected and main genes involved in primary CoQ_10_ deficiencies according to different stages of life. The different *COQ* genes involved and the organs affected are shown. Primary CoQ_10_ deficiency occurs either as a multisystemic disease or by affecting a single organ; in this latter case, genes are indicated in bold. IUGR: intra-uterine growth retardation. Created in BioRender. https://BioRender.com/, accessed on 20 December 2025.

Although dietary intake contributes some CoQ_10_, tissue concentrations depend predominantly on the endogenous biosynthetic pathway described in Section 3 of this review. This is illustrated by the fact that recessive pathogenic variants in 11 of the 14 genes involved in this pathway can lead to primary CoQ_10_ deficiency. In this section, we focus exclusively on the symptoms and organs affected by these primary deficiencies in humans to help clinicians and geneticists consider primary CoQ_10_ deficiency when relevant signs are present. Because mitochondria are found in most human cell types and CoQ_10_ participates in multiple cellular functions, it is not surprising that primary CoQ_10_ deficiencies cause damage in numerous organs, particularly those with high energy demands, as depicted in Figure 3. As we will show, symptoms may manifest during the prenatal period, during infancy, during childhood, or in adulthood.

### 4.1. Antenatal and Neonatal Presentations

Antenatal manifestations generally include intrauterine growth retardation (IUGR), oligohydramnios, cardiac abnormalities (such as cardiomegaly), cerebral anomalies (lissencephaly, thin corpus callosum, cerebellar hypoplasia), renal cysts, intestinal abnormalities (echogenic bowel with dilatations), reduced fetal movements, limb abnormalities, arthrogryposis, and cystic hygroma. Bi-allelic pathogenic variants have been reported in *COQ2* [75], *COQ4* [76], *COQ7* [77,78], and *COQ9* [79]. Brain autopsies have shown neuronal loss with astrocytosis in the brainstem, basal ganglia, and thalami, features suggestive of Leigh disease, as well as damage to the cortex, cerebellum, and white matter [76,77] and lesions indicative of multifocal global ischemic events [79]. Antenatal presentations are typically associated with severe disease, not necessarily due to more deleterious variant types (e.g., nonsense versus missense), and often result in death during the neonatal period or early childhood.

Patients presenting symptoms in the neonatal period have been described with mutations in *COQ2* [44,80,81], *COQ4* [82,83,84], *COQ6* [85], *COQ7* [77,86], *COQ9* [87,88], and *HPDL* [89,90,91,92,93]. These cases typically involve multisystemic disease with neurological features (hypotonia, epilepsy, dystonia, opisthotonos, encephalopathy), cardiac involvement (cardiomyopathy, cardiomegaly, bradycardia, tachycardia, cardiogenic shock), respiratory complications (apnea, respiratory distress), muscular contractures, and less frequently renal abnormalities (proteinuria, tubulopathy, edema). Feeding difficulties, microcephaly, and developmental delays are also common. These presentations are generally extremely severe, with reported deaths occurring from a few hours after birth to 3.5 years of age, although some children were still alive at the time of publication [85,92].

### 4.2. Involvement of the Central Nervous System

Damage to the central nervous system (CNS) is likely the most frequently reported manifestation in patients carrying pathogenic variants in *HPDL*, *PDSS1*, *PDSS2*, *COQ2*, *COQ4*, *COQ5*, *COQ6*, *COQ7*, *COQ8A*, and *COQ9*. The associated neurological symptoms vary widely and may occur in combination with one another or alongside involvement of other organ systems. Epilepsy, described under terms such as infantile spasms, jerks, myoclonus, or epileptic encephalopathy, is commonly observed in patients with mutations in *COQ2* [80,81,94,95], *COQ4* [76,82,83,84,96], *COQ8A* [97,98], *COQ9* [87,88,99,100], and *HPDL* [89,90,91,92,93], and is reported less frequently in *COQ5* [101], *COQ6* [102], *COQ7* [103], and *PDSS1* [104]. Reported prevalence estimates include 32% for *COQ8A,* around 50% for HPDL [92], and more than 70% for *COQ4*, though these values vary depending on the gene and cohort size [84,97].

Epilepsy may appear as early as the neonatal period, as reported for *COQ2* [80,81], *COQ4* [76,83], *COQ9* [100], and *HPDL* [93], or may manifest later, as observed in individuals with *COQ6* mutations [102]. In some cases, seizures are resistant to antiepileptic drugs, such as in COQ9-related disease [87] and *PDSS2* deficiency [105], and can contribute to early mortality [100]. Epilepsy is generally not an initial symptom in infantile or adult-onset forms associated with *COQ4* [96], *COQ7* [86], or *COQ8A* mutations [97], nor in cases where the CNS is not primarily affected, as shown for *COQ2* [94].

Ataxia (also referred to as imbalance disorder) is the initial and predominant feature in patients with *COQ8A* mutations. It is present in all individuals with infantile-onset disease [97], with a moderate overall progression that leads to the need for walking aids or a wheelchair in approximately half of the patients. Additional neurological symptoms may accompany the ataxia. Cerebral imaging consistently shows cerebellar atrophy involving both the vermis and the hemispheres in nearly all affected individuals [97]. Ataxia has also been reported, though less frequently, in patients with other primary CoQ_10_ biosynthesis defects. This includes individuals with *COQ2* mutations presenting with infantile-onset disease associated with tremor and cerebellar atrophy [95] or with adult-onset disease [106]; a young patient with a *COQ4* mutation who developed a spastic-ataxic gait as the initial symptom [96]; three sisters carrying a *COQ5* tandem duplication involving the last four exons, who exhibited non-progressive cerebellar ataxia with dysarthria, nystagmus, and mild cerebellar atrophy [101]; a patient with an infantile-onset *COQ7* mutation [107]; and individuals with *HPDL* mutations, although in the latter ataxia is not a major feature [89].

Spastic paraplegia is a frequent manifestation in patients with *HPDL* mutations. It is typically accompanied by additional neurological features, including intellectual impairment, motor developmental delay, central respiratory failure, and epilepsy, and may present with neonatal, infantile, or juvenile onset. The clinical course is characterized by progressively worsening spasticity and evidence of brain or cortical atrophy, consistent with a neurodegenerative process [89,90,91,92]. Spasticity has been reported much less frequently in association with other primary CoQ_10_ biosynthesis defects. Cases include patients with *COQ2* pathogenic variants, in whom neurological signs such as jerks and brisk reflexes occurred alongside hypertrophic cardiomyopathy [81]; individuals with *COQ4* mutations presenting with infantile-onset disease where spastic paraplegia is the predominant feature [83,96,108]; patients with *COQ7* mutations showing infantile [95] or juvenile onset accompanied by epilepsy and distal amyotrophy [103,109]; and two adult siblings with *COQ9* mutations who developed pure spastic paraplegia with childhood onset [68].

Cervical or focal dystonia affecting the upper limbs has been reported in patients with *COQ8A* mutations. In the cohort described by Traschütz et al., dystonia occurred in approximately 30% of patients with ataxia and often represented the initial presenting symptom [97]. Dystonia has also been described in individuals with *COQ2* mutations, including a pair of dizygotic twins in whom it was accompanied by additional neurological impairments [80].

Other neurological symptoms have been sporadically reported. Stroke-like episodes were described in a young patient with a *COQ2* mutation who initially presented with proteinuria [94], and in 4 out of 59 patients with *COQ8A* mutations [97]. Motor delay, motor deterioration, or absence of psychomotor development has been frequently observed in individuals with *HPDL* mutations [89,91,92,93], in *COQ8A* deficiency [97], in patients with *COQ7* mutations [77,78,95,103,107], with *COQ5* mutations [101], and in one patient with a *COQ4* mutation [83].

Cognitive symptoms, including cognitive deterioration, mild to moderate cognitive disability or delay, intellectual disability, cognitive impairment, mental retardation, language delay, tremor, migraine, and behavioral disorders, have also been reported in patients with *COQ4* [83,96], *COQ5* [101], *COQ8A* (affecting 25–50% of patients) [97], *HPDL* (affecting 50–65% of patients) [89,91,92], and *PDSS1* mutations [110,111].

Encephalopathy has been described in both infantile- and adult-onset *COQ2* deficiency [44], in neonatal-onset *COQ4* deficiency [83], and in 35–65% of patients with *HPDL* mutations [89,90], with a predominance (up to 85%) in individuals presenting with a severe phenotype [89]. It has also been reported in a patient carrying both a deletion and a mutation in *PDSS2* [112].

Brain imaging reveals a wide range of lesions and abnormalities affecting the CNS. Cerebellar atrophy or hypoplasia, often involving the vermis, is among the most frequently reported findings in patients with pathogenic variants in the CoQ_10_ biosynthetic pathway. It affects approximately 95% of individuals with *COQ8A* mutations [97], around 15% of those with *HPDL* mutations [89,91], and has also been described in patients with mutations in *COQ2* [75,94,95,106], *COQ4* [76,83,84], *COQ5* [101], and *COQ9* [87,99]. White matter abnormalities, described as white matter changes, leukomalacia, leukoencephalopathy, or delayed myelination, are also common across several genetic forms of primary CoQ_10_ deficiency. These findings have been reported in individuals with mutations in *COQ2* [95], *COQ4* [84], *COQ6* [102], *COQ7* [77,86,103,107], *HPDL* [89], *PDSS1* [104,113,114]. In the cohort described by Wiessner et al., white matter abnormalities were present in approximately 40% of *HPDL*-mutated patients, predominantly among those with a severe phenotype [89]. Symmetrical lesions in the basal ganglia and brainstem, reminiscent of Leigh syndrome, have been reported in patients with mutations in *COQ4* [84], *COQ7* [77], *COQ9* [88], *HPDL* [89,115], *PDSS1* [104], and PDSS2 [105]. Additional reported abnormalities include agenesis or hypoplasia of the corpus callosum [78,89,99], brainstem hypoplasia [78,96,99], cortical abnormalities, and generalized cerebral atrophy [81,84,86,87,89,90,91,94].

### 4.3. Involvement of the Peripheral Nervous System

Peripheral polyneuropathy, or distal hereditary motor neuropathy, is frequently reported in patients with *COQ7* mutations [57,77,109,116,117,118]. Symptoms typically begin in childhood or adolescence and manifest as difficulties with running or walking, muscle weakness, and frequent falls. These symptoms gradually worsen and progress toward amyotrophy. Foot deformities, such as *pes cavus* or hammer toes, are also observed. Deep tendon reflexes may be normal, reduced, or brisk. Nerve conduction studies usually show an axonal motor neuropathy, although sensory involvement has also been reported. Peripheral neuropathy has additionally been described in two siblings with a *PDSS1* mutation [110] and in a patient with a *COQ2* mutation [95]. In one case, sural nerve biopsy revealed mild mixed axonal and demyelinating degeneration [109].

### 4.4. Involvement of the Muscular System

Skeletal muscle involvement has been well documented in patients carrying mutations in *COQ2* [80,81,94,95], *COQ4* [76,82,83,84], *COQ5* [119], *COQ6* [102], *COQ7* [78,86,107], *COQ8A* [97], *COQ9* [87,88,99,100], *HPDL* [89], *PDSS1* [113], and *PDSS2* [105]. Muscle involvement is rarely isolated and more commonly occurs alongside neurological or cardiac manifestations. Although early signs may appear during the antenatal period, typically as decreased fetal movements [76], most symptoms have neonatal or infantile onset. In the neonatal period, hypotonia, when present, is frequently accompanied by respiratory difficulties or distress and feeding problems [76,80,81,83,84,86,87,88,113]. Additional findings reported at birth include arthrogryposis [83,100], reduced spontaneous movement, and progressive decline in muscle tone [82]. When muscle symptoms emerge during childhood or adolescence, they primarily present as muscle weakness [95,111,119], exercise intolerance [97], walking difficulties [107], or difficulty maintaining head control [105], with gradual progression over time. In late-onset cases, muscle involvement is rarely the initial symptom but may affect approximately 25% of individuals with *COQ8A* mutations [97]. Other less frequently reported manifestations include oculomotor abnormalities [89], dysphagia and ptosis [97], and elevated creatine phosphokinase (CPK) levels [95]. Histological and histo-enzymatic analyses have been performed in a limited number of studies using muscle biopsy samples and have shown heterogeneous findings. Reported abnormalities include lipid accumulation [78,120] and subsarcolemmal mitochondrial proliferation, identified using Gomori’s trichrome staining or succinate dehydrogenase labeling [78,94,105,120,121]. Cytochrome *c* oxidase–negative fibers have also been described [120,121]. In some cases, muscle biopsy results are normal [117] or reveal only subtle abnormalities, such as an increased proportion of type 2C fibers [101]. Electron microscopy may show mitochondria that are abnormal in size or structure or otherwise damaged [78,117].

### 4.5. Involvement of the Heart

Cardiac involvement has been reported in patients carrying mutations in *COQ2*, *COQ4*, *COQ6*, *COQ7*, *COQ9*, *PDSS1*, and *PDSS2*, with presentations occurring in the antenatal period [83,84,88], the neonatal period [76,77,78,79,81,82,83,84,86,100], or during infancy [99,107,111,112,113]. Cardiac manifestations are rarely isolated and instead typically occur within a multisystemic presentation that also involves the renal, neurological, muscular, ocular, and auditory systems. Echocardiographic findings include hypertrophic or dilated cardiomyopathy, systolic or diastolic dysfunction with reduced ejection fraction involving the right ventricle, the left ventricle, or both, left ventricular non-compaction cardiomyopathy, atrial or ventricular septal defects, tricuspid or mitral regurgitation, mitral dysplasia, and pulmonary hypertension. Cardiac rhythm disturbances, such as arrhythmia, bradycardia, and ventricular tachycardia, have also been reported. These abnormalities commonly progress to heart failure, cardiogenic shock, and early death in most cases. Autopsy findings, reported in a limited number of patients, include biventricular hypertrophy, left ventricular hypoplasia with septal hypertrophy, cardiomyocyte hypertrophy and vacuolization, necrosis, endocardial fibrosis, edema, and lymphocytic infiltration [76,82,83].

### 4.6. Involvement of the Kidneys

Interestingly, it is possible to distinguish isolated renal forms, seen almost exclusively in patients with *COQ8B* mutations [122,123,124,125,126], from multisystemic forms in which renal involvement co-occurs with abnormalities in other organs such as the brain, eyes, skeletal muscle, and heart. These multisystemic presentations are associated with mutations in *COQ2* [44,81,94,127], *COQ6* [85,102,128,129,130], *COQ7* [77,78,86], *COQ9* [79,87,99,100], *PDSS1* [104,114], and PDSS2 [105,112], and frequently lead to a fatal outcome. Renal disease typically manifests as proteinuria of varying severity, sometimes associated with edema and arterial hypertension, tubulopathy, acute kidney injury, or a steroid-resistant nephrotic syndrome, which often progresses to end-stage renal failure and anuria. Although renal symptoms most commonly appear during childhood, adolescent- and adult-onset forms have also been reported. Earlier presentations include antenatal manifestations such as oligohydramnios and hyperechoic kidneys [78,79] and prenatal findings such as tubular dysfunction, hyperechoic kidneys, and renal cysts [87,100]. A large Chinese study of 120 children with proteinuria or focal segmental glomerulosclerosis associated with steroid-resistant nephrotic syndrome identified *COQ8B* as the most frequently mutated gene [124]. When kidney biopsy is performed, histological analysis almost invariably reveals focal segmental glomerulosclerosis [81,112,122,125,126,128,131], characterized by mesangial hypercellularity, expansion of the mesangial matrix, sclerosis of glomerular capillaries, thickening of the glomerular basement membrane, foot-process effacement, enlarged podocytes, and an increased number of swollen or structurally abnormal mitochondria.

### 4.7. Involvement of the Eyes

Eye involvement, although less frequently reported than other organ manifestations, has been described in patients with mutations in *COQ2* [80,94,119,127,132], *COQ4* [108,119], *COQ5* [119], *COQ6* [129], *COQ8A* [97], *PDSS1* [110,111,113,119], and *PDSS2* [112]. A wide spectrum of ophthalmological findings has been reported, including vision loss, night blindness, photophobia, optic disc pallor, central scotoma, abnormal color vision, cataract, retinal dystrophy, macular edema, pigmentary retinopathy, and optic atrophy. Among these, pigmentary retinopathy and optic atrophy are the most frequently observed and may occur together, as reported by Traschütz et al. in a patient with a *COQ8A* mutation [97]. Isolated ocular involvement is rare. One exception is the case described by Kurata et al., in which a 20-year-old patient presented with night blindness, photophobia, and progressive vision loss associated with pigmentary retinopathy, without another organ involvement [132]. In most other reports, eye abnormalities occur in the context of multisystem disease and are associated with neurological, renal, muscular, or cardiac manifestations, or with hearing loss. In such multisystemic cases, ocular symptoms are generally not the initial presentation and tend to develop during childhood, adolescence, or even adulthood.

### 4.8. Involvement of the Auditory System

Bilateral sensorineural hearing loss has been reported in patients with mutations in *COQ6* [129,130], *COQ7* [77,78,86,95], *PDSS1* [110,111,113], and *PDSS2* [112]. Although hearing loss is occasionally isolated [130], it is more commonly observed as part of a multisystemic presentation, associated with neurological, muscular, renal, cardiac, ophthalmological, or gastrointestinal involvement. Deafness may be detected at birth through neonatal hearing screening [112], during the neonatal period [77,78,86], or appear later in childhood [95,110,111,113,129,130]. Sensorineural impairment is documented using audiometry or brainstem auditory evoked response testing.

### 4.9. Involvement of Other Organs

Damage to other organs has been reported, although only in very isolated cases. Pulmonary arterial hypertension has been observed in patients with mutations in *PDSS1* [110,113] and *PDSS2* [112]. Regarding the digestive system, Pettenuzzo et al. described meconium peritonitis in a two-day-old newborn carrying mutations in the *COQ7* gene, associated with intestinal atresia that required multiple surgical interventions. Hepatic involvement has also been reported [78]. Wang et al. described hepatomegaly as part of a multisystem presentation (cardiac, renal, and pulmonary involvement) in a 3-month-old infant with *COQ6* mutations [102]. Additionally, Mollet et al. reported liver failure in a 2-day-old girl with *PDSS1* mutations, associated with neurological and renal abnormalities [110].

## 5. Roles of Coenzyme Q_10_ and Pathophysiological Hypotheses

At first glance, it may seem surprising that the same biochemical abnormality, namely CoQ_10_ deficiency caused by pathogenic variants in genes involved in its biosynthesis, can result in a wide spectrum of clinical phenotypes, ranging from isolated organ involvement to multisystemic disease, despite the ubiquitous nature of CoQ_10_. In primary CoQ_10_ deficiency, a reduction in total cellular CoQ_10_ levels has been consistently demonstrated in patient-derived tissues, particularly muscle and fibroblasts, suggesting a global depletion affecting both mitochondrial and non-mitochondrial compartments; however, this has never been clearly demonstrated.

This observation raises the possibility that multiple cellular processes dependent on CoQ_10_ are disrupted and contribute to disease pathophysiology. However, an important question remains: why are certain tissues preferentially affected, whereas others appear relatively spared? One intuitive hypothesis is that tissues with high energy demand may be more vulnerable to CoQ_10_ depletion, given the central role of this cofactor in mitochondrial ATP production. Supporting this notion, pathogenic variants in *COQ8B* predominantly lead to renal manifestations such as proteinuria, steroid-resistant nephrotic syndrome, or focal segmental glomerulosclerosis (see Section 4.6). Nevertheless, this explanation alone is insufficient, as other highly energy-dependent organs, such as the heart, skeletal muscle, or central nervous system, are preserved in these isolated forms, even though they are commonly involved in mitochondrial disorders. This discrepancy suggests that the pathophysiological mechanisms underlying primary CoQ_10_ deficiencies cannot be fully explained by impaired energy production alone.

The following section details the various physiological functions of CoQ_10_ within both mitochondrial and non-mitochondrial compartments. Building on these pathways, we will categorize the putative pathophysiological mechanisms responsible for the tissue and organ alterations described in Section 4 in patients harboring deleterious variants in *COQ* genes. We have ranked these processes as major, moderate, or minor based on the volume of existing literature, the consistency of results across experimental models, and the successful reversal of anomalies through pharmacological or genetic interventions. Mechanisms are classified as “major” when they have been consistently observed across multiple experimental models, are supported by a substantial body of literature, and demonstrate reversibility upon the normalization of CoQ_10_ levels. Mechanisms were categorized as moderate if they had been demonstrated in only a single experimental model or if existing data remained contradictory. Finally, we defined mechanisms as minor when they were purely hypothetical or lacked support from robust experimental data in the literature.

Various experimental models have been utilized to characterize CoQ_10_ biosynthesis and the mechanisms underlying cellular alterations in primary deficiencies. While these models offer distinct advantages, they also present limitations regarding their translatability to human physiology and pathology. For instance, the yeast model is frequently used to identify biosynthetic enzymes due to the ease of gene inactivation and its clear phenotype. However, while most yeast *Coq* genes have mammalian homologs, some possess multiple counterparts, and the overall homology between yeast and human orthologs is relatively low. Conversely, patient-derived dermal fibroblasts are widely used to validate the pathogenicity of *COQ* variants and study cellular abnormalities. These provide a human tissue model that is easily cultured and obtained through minimally invasive skin biopsies. Furthermore, these models are highly amenable to the employed techniques, allowing for the modulation of protein activity or expression through both pharmacological and genetic approaches. While human tissues such as muscle, liver, and heart are used for diagnostic purposes, their utility in characterizing pathophysiological mechanisms is limited by small sample sizes and the requirement for immediate processing. To address these limitations, preclinical models, including knockout (KO) or knock-in (KI) mice, have been developed to simulate human mutations. These models offer the advantage of studying organ and cellular alterations within a complex, physiological environment that closely mirrors human pathology. By integrating these findings with clinical and biochemical data, it is possible to establish a framework for understanding the tissue specificity and phenotypic variability observed in primary CoQ_10_ deficiencies. Furthermore, induced pluripotent stem cells (iPSCs) represent a novel approach to studying mutations in a tissue-specific context. Derived from patient fibroblasts, iPSCs can be differentiated into neurons, cardiomyocytes, or myocytes and cultured in 2D or 3D systems to create complex organoid models. While this approach has been applied to mitochondrial diseases and specifically to *COQ2* and *COQ4* variants, some findings remain surprising [133]. For instance, Romero-Moya et al. observed cellular alterations in iPSC-derived neurons carrying a heterozygous *COQ4* variant, which is unexpected given the autosomal recessive nature of CoQ_10_ deficiencies [134]. Similarly, Nakamoto et al. studied the impact of two *COQ2* variants in iPSC-derived neurons, though one is classified as a common polymorphism in the Japanese population [135].

### 5.1. Involvement of Coenzyme Q_10_ in Energy Metabolism

#### 5.1.1. Mitochondrial Respiration and ATP Production

**Figure 4:**
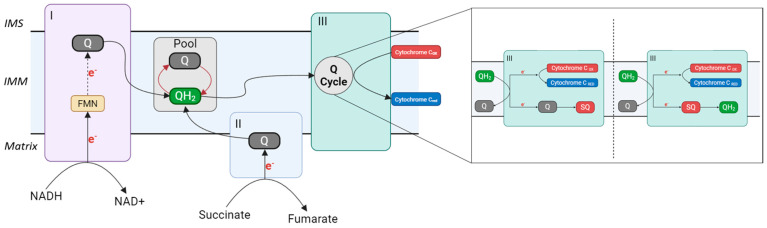
Coenzyme Q_10_ in the mitochondrial respiratory chain. IMM: inner mitochondrial membrane; IMS: intermembrane space; I: complex I; II: complex II; III: complex III; Q: Ubiquinone; QH_2_: Ubiquinol. SQ: semi-quinone. Created in BioRender. https://BioRender.com/, accessed on 20 December 2025.

Since its discovery in the 1960s, the best-characterized role of CoQ_10_ is its function as a mobile electron carrier within the MRC. CoQ_10_ shuttles electrons from complexes I and II, as well as from several other dehydrogenases, to complex III, thereby sustaining the proton motive force that drives ATP synthesis [136,137]. Complex I (NADH-CoQ oxidoreductase) is a flavoprotein that transfers two electrons from NADH to CoQ_10_ via flavin mononucleotide (FMN) and eight Fe–S clusters. This transfer is coupled to the translocation of protons across the IMM [138]. Complex II (succinate-CoQ oxidoreductase) participates in both the MRC and the Krebs cycle, where it catalyzes the oxidation of succinate to fumarate with the concomitant reduction of FAD to FADH_2_. The two electrons derived from this reaction are transferred to CoQ_10_, reducing it to CoQH_2_ via three Fe–S clusters. Unlike complex I, this process does not contribute directly to proton pumping [139]. Once reduced, CoQH_2_ diffuses freely within the lipid bilayer of the IMM to deliver its electrons to complex III (CoQ–cytochrome *c* oxidoreductase). Electron transfer through complex III follows a circular pathway known as the Q cycle, which ensures the reoxidation of CoQH_2_, the reduction in two cytochrome *c* molecules, and the net pumping of protons [140]. Complex IV (cytochrome *c* oxidase) catalyzes the terminal step of the MRC by transferring electrons from reduced cytochrome *c* to molecular oxygen, while concomitantly pumping additional protons across the IMM [141]. The resulting proton gradient powers complex V (ATP synthase), ensuring efficient ATP production (Figure 4) [142].

The precise organization underlying mitochondrial electron transfer has long been the subject of debate, gradually evolving from the classical “random collision model” toward more structured conceptual frameworks [143]. According to the random collision model, the entire CoQ_10_ pool functions as a homogeneous reservoir accessible to all mitochondrial respiratory complexes and dehydrogenases. This view is supported by evidence that the majority of mitochondrial CoQ_10_ (approximately 70–90%) is not tightly bound to membrane proteins. It posits that respiratory chain complexes, dehydrogenases, and CoQ_10_ diffuse freely and independently within the IMM, with electron transfer occurring through random collisions between CoQ_10_ and its enzymatic partners [144]. The subsequent discovery of respiratory supercomplexes (SCs) introduced a more organized paradigm, wherein individual complexes I, III, and IV can assemble into stable supramolecular entities, most prominently CI/CIII_2_, CIII_2_/CIV, and CI/CIII_2_/CIV [145]. In this model, each SC is proposed to sequester a specific subpopulation of CoQ_10_ molecules, thereby enhancing electron transfer efficiency through substrate channeling [146]. Indeed, isolated SCs have been shown to be catalytically competent, capable of sustaining electron transfer without requiring exogenous CoQ_10_. These findings gave rise to the contemporary “plasticity model,” which reconciles both perspectives by proposing a dynamic equilibrium between freely diffusing complexes and diverse SC assemblies [147]. Within this framework, the CoQ_10_ pool is no longer viewed as entirely homogeneous but rather as dynamically partitioned into at least two functionally distinct fractions: (1) a subset of CoQ_10_ molecules associated with or sequestered by SCs, facilitating efficient, channeled electron transfer from NADH (the CoQNADH pool); and (2) a freely diffusing pool within the IMM that serves complex II and other dehydrogenases (the CoQFADH_2_ pool) [143]. Despite this apparent compartmentalization, kinetic data indicate that these CoQ_10_ subpools remain interconnected, such that the overall CoQ_10_ population operates as a single functional continuum in which all dehydrogenases ultimately compete for the same redox mediator.

Given the role of CoQ_10_ in mitochondrial bioenergetics, a primary deficit of this cofactor produces a reduction in complexes I+III, II+III, and GPD (glycerol-3-phosphate dehydrogenase)+III activities, a decrease in oxygen consumption rate and variable impairments in ATP synthesis. Studies on tissues derived from patients carrying mutations in genes involved in CoQ_10_ biosynthesis (including *COQ2*, *COQ4*, *COQ7*, *COQ8B*, *HPDL* and *PDSS1*) have demonstrated variable impact on mitochondrial respiration and/or on ATP synthesis [57,80,83,90,101,107,122,148]; these differences in results are likely related to the varying impact of pathogenic variants, the level of CoQ_10_ depletion, and the tissues analyzed. On muscle biopsy, Jakobs and collaborators also demonstrated a decrease in pyruvate oxidation and in ATP production associated with CoQ_10_ depletion [80]. In fibroblasts, a correlation between CoQ_10_ level and the reduction in maximal mitochondrial respiration is observed among different patients [83]. Some studies observed that the decrease in CoQ_10_ fibroblast content only reduced the maximal respiration rate [83,107,122], while others showed that the basal respiration was also altered in lymphocytes [90] and in fibroblasts [57]; such differences can be explained by different techniques or experimental conditions used for these measurements. Alongside the decrease in CoQ_10_, a reduction in ATP synthesis is observed by some in muscle [80] and fibroblasts [57] but not by others [107]. These discrepancies can be explained by the ATP assay, which is not a robust technique, and by the use of different substrates to fuel the respiratory chain. It is also noteworthy that supplementation of patient’s fibroblasts with CoQ_10_ rescues mitochondrial respiration [57,68,107].

Experiments performed on patient-derived fibroblasts cultured in galactose medium, which forces reliance on oxidative phosphorylation for energy production, have shown a strong dependence of ATP levels on the severity of CoQ_10_ deficiency. While a major deficiency in CoQ_10_ (≤40% of normal values) leads to a significant decrease in ATP levels, this is not the case with moderate deficiency (up to 50% of normal values) [149]. This energy deficit could contribute, at least in part, to the multisystemic clinical manifestations characteristic of primary CoQ_10_ deficiencies.

In conclusion, based on the current evidence and numerous studies demonstrating a correlation between CoQ_10_ depletion and impaired mitochondrial respiration and ATP synthesis, disruption of energy metabolism appears to be a major contributor to the pathogenesis of primary CoQ_10_ deficiency, particularly in high-energy-demand organs such as the heart, liver, skeletal muscles, kidneys, and neurons. Overall, the severity of CoQ_10_ deficiency generally correlates with the degree of impairment in the respiratory chain and ATP production. In vitro supplementation of patient-derived cells with CoQ_10_ can often restore mitochondrial respiration and ATP synthesis.

#### 5.1.2. Coenzyme Q_10_ in Mitochondrial β-Oxidation

**Figure 5:**
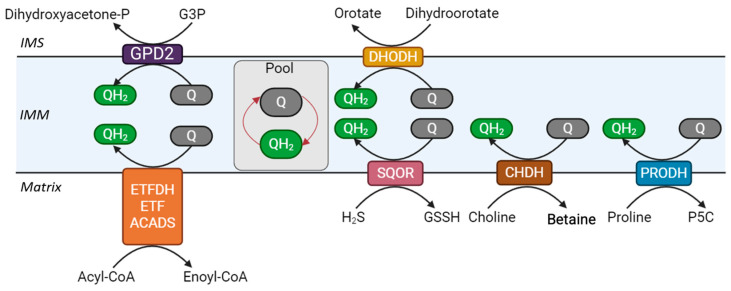
Major enzymatic reactions using coenzyme Q_10_ as an electron acceptor outside the mitochondrial respiratory chain. GPD2: Mitochondrial glycerol-3-phosphate dehydrogenase; ETFDH: Electron-transferring flavoprotein dehydrogenase (ETF–ubiquinone oxidoreductase); ETF: Electron-transferring flavoprotein; ACADS: Short-chain acyl-CoA dehydrogenase; DHODH: Dihydroorotate dehydrogenase; SQOR: Sulfide:quinone oxidoreductase; CHDH: Choline dehydrogenase; PRODH: Proline dehydrogenase. Created in BioRender. https://BioRender.com/, accessed on 20 December 2025.

Electron-transferring flavoprotein dehydrogenase (ETFDH), also known as ETF-QO (electron transfer flavoprotein–ubiquinone oxidoreductase), is a mitochondrial inner membrane enzyme that plays a pivotal role in funneling electrons into the CoQ_10_ pool (Figure 5). ETFDH receives electrons from electron-transfer flavoprotein (ETF), which in turn is reduced by at least nine distinct acyl-CoA dehydrogenases involved in mitochondrial β-oxidation of fatty acids and in the catabolism of branched-chain amino acids [150]. Pathogenic variants in ETFDH cause multiple acyl-CoA dehydrogenase deficiency (MADD), a disorder characterized by impaired fatty acid oxidation and prominent muscle manifestations, including fatigue, exercise intolerance, and myopathy without [151] or with CoQ_10_ deficiency [152].

Whether primary CoQ_10_ deficiencies directly impair the ETFDH pathway remains unclear. However, accumulating evidence suggests that CoQ_10_ deficiency is associated with a broader disruption of lipid metabolism. Patients carrying pathogenic variants in genes involved in CoQ_10_ biosynthesis, such as *COQ2*, *PDSS2*, and *ADCK2*, frequently display metabolic signatures reminiscent of MADD, including muscular impairment (see Section 4.4). In individuals with ADCK2 haploinsufficiency, as well as in the Adck2^+^/^−^ mouse model, moderately increased plasma levels of saturated short- and medium-chain acylcarnitines have been reported, consistent with impaired fatty acid oxidation and reduced electron transfer to the CoQ_10_ pool. Adck2^+^/^−^ mice also show significantly increased urinary excretion of adipic and ethylmalonic acids, together with reduced circulating β-hydroxybutyrate and free carnitine levels [65]. Alterations in urinary organic acids are commonly observed in mitochondrial diseases, including increased excretion of tricarboxylic acid (TCA) cycle intermediates, ethylmalonic acid, and 3-methylglutaconic acid [153]. A patient with a fatal neonatal *COQ2* mutation displayed mild dicarboxylic aciduria and slightly elevated alanine [154]. Similarly, three individuals from unrelated families with pathogenic *COQ4* variants showed elevated 2-hydroxyglutarate levels, supporting ETFDH deficiency [83]. A major consequence of these alterations is lipid accumulation in tissues with high energetic demand, particularly skeletal muscle. Lipid storage disorder has been documented in muscle biopsies from patients with neonatal-onset *COQ2* deficiency and from individuals with *ADCK2* haploinsufficiency, both displaying mitochondrial myopathy with prominent lipid droplets [155]. Lipid accumulation was also observed in patients with pathogenic variations in *COQ7* [78] and *COQ8A* (see Section 4.4) [120]. These findings are recapitulated in the Adck2^+^/^−^ mouse model, which exhibits marked lipid accumulation in skeletal muscle as well as hepatic steatosis, reflecting a systemic defect in lipid metabolism driven by insufficient CoQ_10_ availability [156]. In addition, Pdss2^kd/kd^ mice show increased levels of short-chain acylcarnitines (C4–C6), which have been attributed to decreased activity of sulfide:quinone oxidoreductase (SQOR), another CoQ-dependent enzyme, although ETFDH function was not directly assessed in this model [157].

In conclusion, although primary CoQ_10_ deficiency may be associated with impaired fatty acid oxidation and lipid accumulation, particularly in muscle and liver, the evidence supporting a direct effect on the ETFDH pathway is limited and inconsistent. Thus, disruption of β-oxidation should be considered a minor contributor to the pathogenesis of primary CoQ_10_ deficiency. In addition, whether the observed lipid alterations primarily result from defective electron transfer through ETFDH, from secondary dysfunction of other CoQ-dependent enzymes such as SQOR, or from a combination of both mechanisms remains to be determined.

#### 5.1.3. Glycerol-3-Phosphate Shuttle

Mitochondrial glycerol-3-phosphate dehydrogenase 2 (GPD2) is a flavin-dependent dehydrogenase that catalyzes the oxidation of glycerol-3-phosphate (G3P) to dihydroxyacetone phosphate (DHAP) while transferring electrons from FADH_2_ to CoQ_10_ (Figure 5). This enzyme functions together with the cytosolic glycerol-3-phosphate dehydrogenase 1 (GPD1) to form the glycerol-3-phosphate shuttle, which regenerates cytosolic NAD^+^ from glycolysis-derived NADH and delivers electrons to the electron transport chain via CoQ_10_. GPD2 is anchored to the inner mitochondrial membrane on the intermembrane-space side [158]. As a key component of intermediary metabolism, GPD2 links glycolysis and oxidative phosphorylation and triglyceride synthesis through the glycerol-3-phosphate shuttle. The activity of GPD2 associated with the respiratory chain is known to vary considerably between tissues. It is highly active in the brain, fibroblasts, and lymphocytes. In contrast, negligible glycerol-3-phosphate dehydrogenase activity has been observed in the heart, kidneys, and liver [158].

GPD2 has been little explored in the context of primary CoQ_10_ deficiency. Rötig et al. demonstrated that CoQ_10_ deficiency directly impairs GPD2 activity when functionally coupled to complex III, assessed as glycerol-3-phosphate:cytochrome *c* reductase (GPD2+III). In lymphocytes from two affected siblings, quinone-dependent activities (CII+III and GPD2+III) were at the lower interval of the control range, while activity ratios (CIV/CII+III and CIV/GPD2+III), which are suggested to be much more sensitive indicators of respiratory chain imbalance, were markedly increased. Addition of decylubiquinone or analogs (idebenone, CoQ_4_, CoQ_6_) led to substantial increases in GPD2+III activity in patient-derived fibroblasts. This quinone responsiveness decreased as mitochondrial CoQ_10_ levels were restored during treatment and increased again upon clinical deterioration. However, whether the CoQ_10_ deficiency is primary or secondary is not indicated by the authors [26].

In conclusion, while CoQ_10_ deficiency may impair GPD2 activity under specific experimental conditions, the available evidence is extremely limited, and the physiological relevance remains unclear. Therefore, alterations of the glycerol-3-phosphate shuttle should be considered a minor contributor to the pathogenesis of primary CoQ_10_ deficiencies.

### 5.2. Involvement of Coenzyme Q_10_ in Non-Bioenergetic Processes

#### 5.2.1. Implication of Coenzyme Q_10_ in Anabolic Pathways

##### Lipids Metabolism

Given the role of CoQ_10_ in the degradation of fatty acids and the involvement of the mevalonate pathway in its synthesis, research has been conducted to better understand the impact of a CoQ_10_ deficiency on lipid metabolism. Lipidomic studies using induced-pluripotent stem cells (iPSC)-derived neurons harboring mutations in CoQ_10_ biosynthesis genes (*COQ2* and *PDSS2*) demonstrate that severe CoQ_10_ depletion (≈12–14% residual levels) leads to a marked repression of mevalonate pathway activity, characterized by a reduced expression of key enzymes such as HMG-CoA reductase (HMGCR) and farnesyl diphosphate synthase (FPPS), along with a decreased availability of lipid precursors including farnesyl- and geranylgeranyl-pyrophosphate [159]. Conversely, moderate CoQ_10_ deficiency (≈80% residual), induced pharmacologically, does not affect enzyme expression of the mevalonate pathway but instead perturbs cholesterol trafficking and storage. These findings indicate that the consequences of CoQ_10_ loss on lipid metabolism depend on the severity of the deficit.

Neurons with CoQ_10_ depletion exhibit a reduction in cholesteryl esters, diacylglycerol, and glycosphingolipids; an increase in phosphatidylglycerol; and dysregulation of lipid efflux mechanisms [159]. Given that lipids play structural and functional roles, particularly in neurons, it is not surprising that the central and peripheral nervous systems are affected.

In conclusion, although CoQ_10_ deficiency was shown to affect lipid metabolism, the current available evidence indicates that these alterations represent a minor contributor to the overall pathogenesis of primary CoQ10 deficiencies. However, given the critical role of lipids in neuronal structure and function, disturbances in lipid homeostasis may play a more pronounced role in neurological manifestations, potentially contributing to the phenotypic variability observed among patients.

##### Pyrimidine Metabolism

Dihydroorotate dehydrogenase (DHODH) is a mitochondrial flavoprotein that catalyzes the oxidation of dihydroorotate to orotate (Figure 5), the fourth and rate-limiting step of the *de novo* pyrimidine biosynthesis pathway leading to the formation of uridine monophosphate (UMP), a precursor of RNA and DNA nucleotides (UTP, CTP, and dTMP) [160]. Located on the outer leaflet of the IMM, DHODH transfers electrons from dihydroorotate via the cofactor FMNH_2_ to CoQ, reducing it to CoQH_2_, which then feeds electrons into complex III of the MRC. This reaction directly couples pyrimidine synthesis to mitochondrial respiration [161].

Loss of respiratory chain function (as in ρ^0^ cells that are depleted in mitochondrial DNA (mtDNA) or in complex III-deficient cells) abolishes DHODH activity, leading to uridine auxotrophy since exogenous uridine bypasses the DHODH-dependent step in pyrimidine synthesis [162]. Similarly, CoQ_10_ deficiency could impair DHODH function by restricting the pool of oxidized CoQ_10_ as an electron acceptor. In line with this idea, fibroblasts harboring *COQ2* mutations with a profound CoQ_10_ depletion (<20% residual CoQ_10_ content) exhibit growth defects that are rescued by exogenous CoQ_10_ or uridine supplementation; the stronger rescue with uridine suggests that impaired nucleotide synthesis, rather than reduced ATP production, is the primary vulnerability in these cells [163]. In galactose medium, the proliferation of fibroblasts from patients with pathogenic *COQ7* and *COQ9* variations was reduced and partially restored by CoQ_10_ supplementation [57,68]. Montero et al. also reported a secondary CoQ_10_ deficiency in both muscle and fibroblasts of a patient with a mtDNA depletion syndrome, suggesting a link between CoQ_10_ deficiency and mtDNA replication [164].

However, severely CoQ_10_-deficient models such as Coq7-knockout or Pdss2/Coq7 double-knockout mouse embryonic fibroblasts grow normally without uridine supplementation, implying that even minimal residual mitochondrial respiration (possibly supported by trace CoQ_10_ present in standard culture medium) is sufficient to maintain DHODH activity [165]. Interestingly, while DHODH mutations cause Miller syndrome with characteristic craniofacial and limb malformations, such developmental defects are not observed in primary CoQ_10_ deficiency. This discrepancy indicates that reduced CoQ_10_ availability impairs DHODH function metabolically but does not recapitulate the profound enzyme inactivation caused by monogenic DHODH loss [166]. Further studies will be needed to determine the extent to which CoQ_10_ deficiency compromises DHODH activity and nucleotide synthesis in different pathological contexts.

In conclusion, CoQ_10_ deficiency can impair DHODH-dependent pyrimidine synthesis, as demonstrated by functional studies in patient-derived fibroblasts, where uridine supplementation rescues growth defects. This position DHODH dysfunction as a moderate contributor to the pathogenesis of primary CoQ_10_ deficiencies. However, results from mouse embryonic fibroblast models suggest that the impact of this mechanism likely depends on the severity of CoQ_10_ depletion and the cellular context, and it may be more pronounced in certain cell types, potentially contributing to the phenotypic variability among patients.

##### Proline Metabolism

Another entry point for electrons into the CoQ_10_ pool involves proline metabolism, mediated by proline dehydrogenase 1 and 2 (PRODH1/2) (Figure 5). PRODH1 catalyzes the oxidation of L-proline to 1-pyrroline-5-carboxylate (P5C), with electrons being transferred directly to CoQ_10_. P5C is subsequently converted into glutamate and then glutamine. PRODH1 plays important roles in energy metabolism, mitochondrial ROS production, and signaling pathways involved in apoptosis and stress responses [167,168]. PRODH2, although closely related to PRODH1, shows a strong preference for trans-4-hydroxy-L-proline as its substrate, a metabolite derived from extracellular matrix collagen turnover. PRODH2 converts hydroxyproline into 3-hydroxy-1-pyrroline-5-carboxylate, which is further metabolized to glyoxylate and pyruvate [169]. Mutations in PRODH1 cause type I hyperprolinemia, an autosomal recessive disorder with highly variable clinical presentation, ranging from asymptomatic individuals to severe neurological and psychiatric manifestations. Hyperprolinemia has not previously been reported in patients with CoQ_10_ deficiency. However, it should be noted that amino acid chromatography is not routinely performed in individuals with primary CoQ_10_ deficiency. Further studies will be needed to determine whether impaired CoQ_10_ availability affects proline and hydroxyproline metabolism [170]. Based on the limited evidence currently available, alterations in proline metabolism should be considered a minor contributor to the pathogenesis of primary CoQ_10_ deficiencies.

##### Choline Metabolism

Choline is an essential nutrient in mammals that serves both as a precursor for the synthesis of membrane phospholipids (via the CDP-choline pathway that produces phosphatidylcholine) and sphingomyelin and for the biosynthesis of the neurotransmitter acetylcholine. A portion of the cellular choline pool is also directed toward the mitochondrial oxidative pathway, where choline dehydrogenase (CHDH), an enzyme located in the IMM and dependent on the CoQ_10_ pool, catalyzes its oxidation to betaine aldehyde, which is subsequently oxidized to betaine by betaine aldehyde dehydrogenase (BADH) (Figure 5) [171]. Betaine then functions as a methyl-group donor in the remethylation of homocysteine to methionine by betaine-homocysteine methyltransferase (BHMT), thereby directly linking choline metabolism to the methionine cycle and to the production of SAM, the universal methyl donor. This pathway is tightly interconnected with the folate cycle, which provides an alternative route for homocysteine remethylation through 5-methyl-THF, and it extends to the transsulfuration pathway, responsible for the synthesis of cysteine, glutathione, and other sulfur-containing metabolites [172]. Thus, through their role in betaine synthesis, choline and the activity of CHDH are involved in redox balance and one-carbon metabolism, and to a lesser extent in membrane integrity and neurotransmission with its implication in phosphatidylcholine and acetylcholine production. At present, it is difficult to conclude that CHDH dysfunction plays a significant role in the pathophysiology of primary CoQ_10_ deficiencies. Indeed, no specific clinical abnormalities attributable to CoQ_10_ deficiency–related CHDH impairment have been demonstrated. Based on the available data, a defect in the remethylation pathway appears more plausible; however, this alteration does not seem to be part of the recognized clinical spectrum of primary CoQ_10_ deficiencies [173]. Finally, CHDH has also been described as a mitophagy sensor [174]. Currently, there is no evidence supporting a reduction in CHDH expression or activity in this context (i.e., CoQ_10_ deficiency), making it difficult to infer an impact on mitophagy. Moreover, its role in mitophagy is independent of its catalytic activity and, therefore, of CoQ_10_ availability. Nonetheless, based on the current available evidence, CHDH-related choline metabolism should be considered a minor contributor to the pathogenesis of primary CoQ_10_ deficiencies while remaining an interesting area for future investigation.

#### 5.2.2. Implication of Coenzyme Q_10_ in Sulfide Metabolism

Another CoQ_10_-linked enzyme is the sulfide:quinone oxidoreductase (SQOR). Sulfide metabolism in mammalian cells involves two main routes: (1) the trans-sulfuration pathway, which produces hydrogen sulfide (H_2_S) from cysteine or homocysteine, and (2) the mitochondrial H_2_S oxidation pathway, which converts H_2_S into sulfate ions (SO_4_^2−^). In mammals, the sulfide oxidation pathway takes place within mitochondria [175]. SQOR, a flavoprotein anchored to the IMM, catalyzes the first and key step of this pathway by coupling the oxidation of H_2_S to the reduction in CoQ_10_. During this process, a persulfide intermediate (E–SSH) is generated on a catalytic cysteine residue of SQOR. This persulfide sulfur is then transferred to glutathione (GSH), the main physiological sulfane sulfur acceptor, yielding glutathione persulfide (GSSH) [176]. GSSH serves as a mobile sulfur carrier that undergoes further oxidation by the iron-dependent persulfide dioxygenase ETHE1, generating sulfite (SO_3_^2−^) and reforming GSH. Alternatively, GSSH can react with sulfite through the sulfurtransferase rhodanese (thiosulfate sulfurtransferase, TST) to produce thiosulfate (S_2_O_3_^2−^), while sulfite can be oxidized to sulfate by sulfite oxidase located in the intermembrane space (IMS) [177]. The final products of this pathway, thiosulfate and sulfate, are excreted, completing the detoxification of H_2_S while contributing to mitochondrial energy metabolism.

In primary CoQ_10_ deficiencies, reduced levels of residual CoQ_10_ destabilize SQOR, leading to its degradation and a marked reduction in enzyme activity [178]. This dose-dependent relationship has been demonstrated both in mammalian models and in human cellular systems. In patient-derived skin fibroblasts carrying pathogenic mutations in various CoQ_10_ biosynthetic genes (including *COQ2*, *COQ4*, *COQ8A*, *COQ9*, and *PDSS2*), a progressive decline in CoQ_10_ content correlates directly with reduced SQOR protein stability and impaired SQOR-dependent respiratory activity. Fibroblasts with residual CoQ_10_ levels below ~30% of normal exhibit the most severe functional impairment, with a marked decrease in oxygen consumption in response to hydrogen sulfide and a pronounced loss of detectable SQOR protein (reduced to ~25–27% of control levels). Conversely, fibroblasts maintaining ~50% of physiological CoQ_10_ show only a partial reduction in sulfide oxidation capacity, indicating that SQOR function is preserved until CoQ_10_ becomes critically limiting as an electron acceptor [157].

A similar threshold effect has been observed in various mouse models of primary CoQ_10_ deficiency, including Coq9^R239X^, Coq9^Q95X^, and Pdss2^kd/kd^. In these animals, the extent of SQOR depletion closely mirrors the local residual CoQ_9_ content and the severity of manifestations in the affected tissue. For instance, in Pdss2^kd/kd^ mice, the kidney, where CoQ_9_ levels drop to ~15%, displays a dramatic reduction in SQOR protein (to ~16% of control) and a near-complete collapse of its activity, while the brain, retaining ~30% of CoQ_9_, maintains SQOR levels comparable to wild-type. Similar results were reported for the two other mouse models; in Coq9^R239X^, the very low level of CoQ_9_ content in different tissues (cerebellum, kidney and muscle) is associated with reduced level of SQOR protein level and activity. In Coq9^Q95X^, where muscles display the most profound CoQ_9_ deficiency by 80% compared to kidney or cerebellum (only 50%), a low SQOR protein content and activity is observed only in muscles [179]. These findings collectively support the existence of a CoQ_10_ threshold, below which SQOR becomes unstable and rapidly degraded. Under these conditions, the oxidation of the mitochondrial H_2_S oxidation pathway is compromised, setting the stage for pathological sulfide accumulation.

Excess H_2_S resulting from this metabolic failure would contribute directly to the cellular dysfunction associated with CoQ_10_ deficiency. H_2_S specifically targets the cytochrome c oxidase (COX) by binding directly to the heme-containing catalytic center that maintains the complex IV in a constant reduced state and accelerates long-term degradation of COX subunits, leading to inhibition of mitochondrial respiration [180]. Elevated sulfide also inhibits short-chain acyl-CoA dehydrogenase (SCAD), leading to impaired β-oxidation of short-chain fatty acids and increased levels of C4–C6 acylcarnitines [178]. Additionally, redox stress induced by H_2_S and oxidized sulfur species depletes antioxidant defenses, particularly glutathione (GSH), through sulfide autoxidation, reduced availability of precursors such as glutamate, and decreased activities of glutathione peroxidase and glutathione reductase [179]. Excess sulfide also enhances protein S-sulfhydration, altering diverse cellular functions, and feeds back on the transsulfuration pathway (modulating H_2_S biosynthesis) with broader consequences for carbon metabolism, including folate cycling, serine biosynthesis, and nucleotide metabolism [181].

Rescue interventions confirm that defective sulfide oxidation is directly caused by CoQ depletion: CoQ_10_ supplementation restores SQOR levels and SQOR-dependent respiration both in vitro in mutant fibroblasts and in vivo in Coq9^R239X^ mice [157,179].

In conclusion, impaired sulfide oxidation due to SQOR instability represents an important and well-supported pathogenic mechanism in primary CoQ_10_ deficiencies. Its contribution appears to be highly dependent on the severity of CoQ_10_ depletion, with a clear threshold below which SQOR becomes unstable and rapidly degraded. Beyond the direct loss of sulfide detoxification capacity, excess H_2_S exerts secondary deleterious effects on mitochondrial respiration through inhibition of complex IV, impairs short-chain fatty acid oxidation via SCAD inhibition, and compromises antioxidant defenses by disrupting glutathione homeostasis. Collectively, these findings position defective sulfide metabolism as a moderate but multifaceted contributor to disease pathogenesis, amplifying mitochondrial dysfunction, particularly in tissues with profound CoQ_10_ depletion.

#### 5.2.3. Implication of Coenzyme Q_10_ in Oxidative Stress

##### Reactive Oxygen Species (ROS), Oxidative Stress and Cellular Toxicity

Oxidative stress arises from an imbalance between the production of ROS, derived from oxygen, and the antioxidant defense systems. In aerobic metabolism, the principal ROS include the superoxide anion (O_2_^−^), hydrogen peroxide (H_2_O_2_), and the hydroxyl radical (HO°) [182]. The two main sources of ROS are mitochondria, as the predominant site of production, and NADPH oxidase in macrophages, which generates those species involved in antimicrobial defense [183,184]. ROS are highly reactive compounds, which interact with multiple cellular components (lipids, proteins, and DNA) and promote their damage, and have substantial cytotoxic effects [185,186,187]. Among all molecular targets, polyunsaturated fatty acids (PUFAs) in mitochondrial and cellular membranes are especially sensitive to oxidation through non-enzymatic lipid peroxidation directly driven by ROS [188]. This process occurs in three main steps:

- Initiation: highly reactive hydroxyl radicals (HO°), often produced via iron-dependent Fenton chemistry from hydrogen peroxide, abstract a hydrogen atom from PUFA chains, generating a lipid radical (L°).
- Propagation: the lipid radical rapidly reacts with molecular oxygen to form a lipid peroxyl radical (LOO°), which then attacks nearby lipids, creating new radicals and sustaining an amplifying chain reaction that produces lipid hydroperoxides (LOOH).
- Termination: the chain reaction stops when two radicals combine to form non-radical products, or when antioxidants such as glutathione break the cycle. The end of this cascade produces reactive secondary aldehydes, notably malondialdehyde (MDA) and 4-hydroxy-2-nonenal (4-HNE), which impair membrane integrity and can form adducts with proteins, DNA, and phospholipids, amplifying oxidative damage [188].

Mitochondrial membranes are especially susceptible to lipid peroxidation because they are enriched in polyunsaturated phospholipids, particularly cardiolipins [189]. Oxidation of cardiolipin alters cristae architecture, destabilizes respiratory SCs, and impairs oxidative phosphorylation, which further increases electron leakage and ROS generation [190]. ROS production triggers a vicious cycle that reinforces and amplifies its own generation. Moreover, it is well established in Barth syndrome that cardiolipin abnormalities promote dysfunction of the mitochondrial respiratory chain [191].

Ferroptosis, first described by Dixon et al. as a regulated, non-apoptotic form of cell death driven by iron-dependent lipid peroxidation, is marked by condensed and shrunken mitochondria, displaying increased membrane density and loss of cristae, while the outer membrane remains intact. Its execution reflects a disequilibrium between pro-oxidant mechanisms responsible for the accumulation of lipid hydroperoxides and the antioxidant systems that normally prevent their lethal conversion into toxic breakdown products [192]. Lyamzaev and colleagues showed a direct relationship between the peroxidation of mitochondrial phospholipids and ferroptosis; mitochondria-targeted antioxidants are powerful inhibitors of this form of cell death [193].

Proteins are also susceptible to oxidative modification that perturbs their structure and function. A predominant mechanism is carbonylation, an irreversible ROS-driven introduction of aldehyde or ketone groups into protein backbones, marking damaged proteins for degradation and contributing to loss of proteostasis when excessively accumulated. Additional modifications on cysteine and tyrosine residues can impair enzyme activity, disrupt redox signaling, and alter protein–protein interactions, ultimately compromising essential metabolic and regulatory pathways [194]. It has been demonstrated in experimental models that mitochondrial proteins undergo oxidative alterations that impair complex I activity and respiration [195]. Finally, ROS can cause extensive damage to the genome, affecting both nuclear and mitochondrial DNA. Oxidative base lesions are particularly frequent, with guanine oxidation producing 8-oxo-7,8-dihydroguanine (8-oxo-dG), a mutagenic lesion capable of inducing G → T transversions if unrepaired. ROS can also break the sugar–phosphate backbone and generate an abasic site through cleavage of the N-glycosidic bond [196]. Mitochondrial DNA, lacking histone protection and located near ROS-producing sites, exhibits especially high vulnerability, leading to mutations, impaired oxidative phosphorylation, and mitochondrial dysfunction. Moreover, secondary aldehydes generated by lipid peroxidation, such as MDA and 4-HNE, can form bulky DNA adducts that hinder replication and repair [197].

##### How Do Mitochondria Generate ROS?

The mitochondrial respiratory chain contains several major sites of ROS production, with complex I (CI) being one of the most prominent. Two ROS-generating sub-sites have been identified within CI: the FMN site and the ubiquinone (CoQ) binding pocket. During the normal oxidation of NADH, electrons are transferred to CoQ; however, a fraction of these electrons may prematurely reduce molecular oxygen to superoxide. This process is greatly amplified when electron flow through CI is restricted or reversed. Under specific metabolic conditions, including a highly reduced ubiquinol (QH_2_) pool coupled to an elevated proton-motive force, electrons can flow backward from QH_2_ to the NAD^+^/NADH pool in the matrix. This phenomenon, known as reverse electron transport (RET), is widely recognized as a potent source of ROS formation, particularly superoxide and its dismutation product, hydrogen peroxide [198,199]. In the present context (i.e., CoQ_10_ deficiency), this mechanism does not appear to be a major contributor, as total CoQ_10_ levels are decreased and the ETC activity is reduced.

Additional ROS originate from other ETC complexes. At complex II (CII), the IIF site of succinate dehydrogenase generally produces minimal ROS under physiological activity, but its contribution markedly increases during dysfunction or when electron flow is restricted. At complex III (CIII), the Qo site within the Q-cycle can generate superoxide through the semiquinone intermediate formed during electron transfer (Figure 4). Notably, superoxide produced at complex III can be released into both the mitochondrial matrix and the IMS. Importantly, the resulting CoQH_2_/CoQ_10_ ratio emerging from these redox interactions functions as a central sensor of respiratory chain activity. This ratio influences mitochondrial SC organization and can promote RET at complex I under highly reduced conditions, thereby amplifying ROS production. Collectively, these mechanisms underscore that CoQ_10_ is not merely a passive electron carrier but a dynamic redox hub that integrates electron flux, structural organization of the respiratory chain, and mitochondrial ROS signaling [200].

Within the IMS, superoxide is rapidly converted into hydrogen peroxide by the Cu/Zn-dependent superoxide dismutase (SOD1), and this hydrogen peroxide can freely diffuse into the cytosol, extending its signaling or damaging effects beyond mitochondria. In the mitochondrial matrix, superoxide is detoxified by the Mn-dependent superoxide dismutase (SOD2 or Mn-SOD), which catalyzes its conversion into hydrogen peroxide, thereby preventing the accumulation of superoxide and limiting oxidative stress within the mitochondrial compartment. In contrast, complex IV (CIV) contributes only marginally to ROS formation due to its high catalytic efficiency in reducing oxygen through a tightly controlled four-electron transfer, minimizing unintended electron leakage [199]. Under normal physiological conditions, the respiratory chain’s ROS output is tightly controlled by antioxidant systems, most notably SODs, which convert superoxide into the more stable and membrane-permeant hydrogen peroxide at moderate levels; H_2_O_2_ functions as a signaling molecule [201]. When an imbalance occurs and ROS production exceeds the capacity of antioxidant defense systems, these reactive species begin to accumulate and interact with cellular constituents and organelles. Under mild oxidative stress, damaged molecules can be repaired or eliminated through quality-control mechanisms, preserving cellular integrity. However, when oxidative damage becomes excessive or irreversible, ROS compromise essential cellular functions and trigger regulated cell death pathways, including apoptosis, necrosis, pyroptosis, and ferroptosis [201].

##### Antioxidant Systems

Cells maintain redox homeostasis through a coordinated set of antioxidant systems that neutralize ROS and prevent oxidative damage. The first line of defense is provided by SODs, which convert superoxide anion into hydrogen peroxide. H_2_O_2_ generated by SODs is then detoxified by glutathione-dependent enzymes [202].

The glutathione (GSH and GSSG) system constitutes a central axis of cellular antioxidant defense. Reduced glutathione (GSH), abundant in the cytosol and mitochondria, directly scavenges ROS and participates as a cofactor for glutathione peroxidases (GPX). GPXs form a family of selenium-dependent antioxidant enzymes that reduce hydrogen peroxide and organic hydroperoxides using glutathione as an electron donor to water or non-toxic alcohols [203]. Several isoforms coexist in mammalian cells with distinct subcellular localizations and functions: GPX1 is widely expressed in the cytosol and mitochondria and primarily detoxifies hydrogen peroxide; GPX2 is enriched in the gastrointestinal epithelium; GPX3 is secreted into the extracellular space and contributes to plasma antioxidant defense; and GPX4 is unique in its ability to directly reduce phospholipid hydroperoxides within biological membranes [204]. Importantly, GPX4 is the only GPX isoform capable of preventing the accumulation of membrane lipid peroxides, making it the sole member of the family essential for the suppression of ferroptosis [205]. It is worthy to note that the efficient GPX4 translation requires the isopentenylation of the selenocysteine tRNA^[Ser]Sec^, coupling the mevalonate pathway to the antioxidant system [206]. Thus, alteration of the mevalonate pathway could also reduce the efficacy of GPX4 (see Section Lipids Metabolism).

The pool of GSH is maintained by glutathione reductase (GR), ensuring continuous regeneration from oxidized glutathione (GSSG). Complementing this system, glutathione-S-transferases (GSTs) catalyze the conjugation of GSH to electrophilic compounds, including toxic lipid peroxidation products, thus facilitating their detoxification and export [207]. Together, these antioxidant systems provide overlapping layers of protection: SODs prevent superoxide accumulation, GPXs eliminate peroxides, GSH provides reducing power, and GR and GST remove oxidized by-products. This multilayered defense not only prevents ROS-induced macromolecular damage but also preserves signaling processes that rely on controlled redox balance.

Its ability to transport electrons also makes CoQ_10_ a strong candidate in the defense against oxidative stress, as it is capable of capturing electrons. Beyond its well-established function within the mitochondrial electron transport chain, CoQ_10_ participates in multiple antioxidant systems designed to counteract ROS. A central component of these systems is the plasma membrane redox system (PMRS), which transfers reducing equivalents from intracellular donors, primarily NADH and NADPH, to extracellular acceptors. Through this process, the PMRS contributes to maintaining the NAD(P)H/NAD(P)^+^ redox balance, a major determinant of metabolic fluxes, redox signaling, and cellular stress responses. Preserving this redox ratio prevents excess electron accumulation, limiting their diversion toward oxygen and thereby avoiding ROS generation [208].

**Figure 6:**
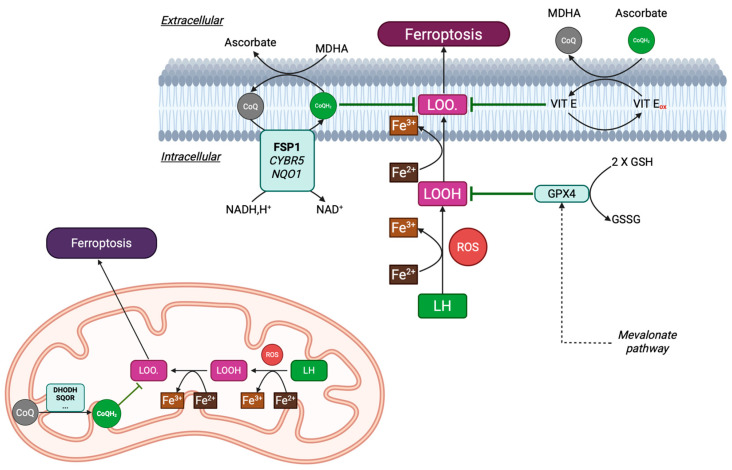
Schematic representation of ferroptosis and the role of coenzyme Q_10_ in its regulation. In the upper part of the diagram, the cellular protective system against ferroptosis is illustrated, involving CoQ_10_ in synergy with vitamin E or tocopherol (radical and reduced) and vitamin C (MDHA: monodehydroascorbate and ascorbate). CoQ_10_ exerts its protective function at two levels. First, in its reduced form (CoQH_2_), it donates electrons to regenerate tocopherol radical (VIT E_ox_) into tocopherol reduced (VIT E) with ascorbate. Second, it directly neutralizes lipid peroxyl radicals (LOO•), a process dependent on its reduction by the FSP1 pathway, which utilizes NADH. Another major anti-ferroptotic defense mechanism is mediated by GPX4, which uses glutathione to reduce lipid hydroperoxides (LOOH) and prevent the accumulation of oxidative lipid damage. In the lower part of the diagram, an analogous mechanism is depicted within mitochondria, where DHODH and/or SQOR provide an additional layer of protection by reducing mitochondrial CoQ_10_, thereby limiting the initiation of ferroptosis in this compartment. Created in BioRender. https://BioRender.com/, accessed on 20 December 2025.

The PMRS comprises several membrane-associated enzymes and small antioxidant molecules, including extramitochondrial CoQ_10_, vitamins C and E. Crucially, three enzymes directly depend on CoQ_10_ as their redox partner: NADH–cytochrome b_5_ reductase (CYB5R), NAD(P)H:quinone oxidoreductase 1 (NQO1), and ferroptosis suppressor protein 1 (FSP1) [209,210,211]. These enzymes, located on the inner leaflet of the plasma membrane, extract electrons from cytosolic NAD(P)H and transfer them to CoQ_10_ embedded in the bilayer [212]. The resulting QH_2_ can then donate electrons either to the tocopherol radical, suppressing lipid peroxidation, or to the monodehydroascorbate radical (MDHA), regenerating ascorbate. Through this dual antioxidant recycling mechanism, CoQ_10_ provides an additional layer of protection against oxidative stress and ferroptosis (Figure 6) [213]. Although GPX4, FSP1, and DHODH all contribute to ferroptosis suppression through the maintenance of reduced ubiquinol, DHODH represents the principal enzymatic defense against ferroptosis within mitochondria [214,215,216], while GPX4 and FSP1 predominantly act in non-mitochondrial membranes [217]. Therefore, ferroptosis protection depends not only on antioxidant activity but also on the compartmentalized reduction of CoQ_10_ into CoQH_2_.

**Figure 7:**
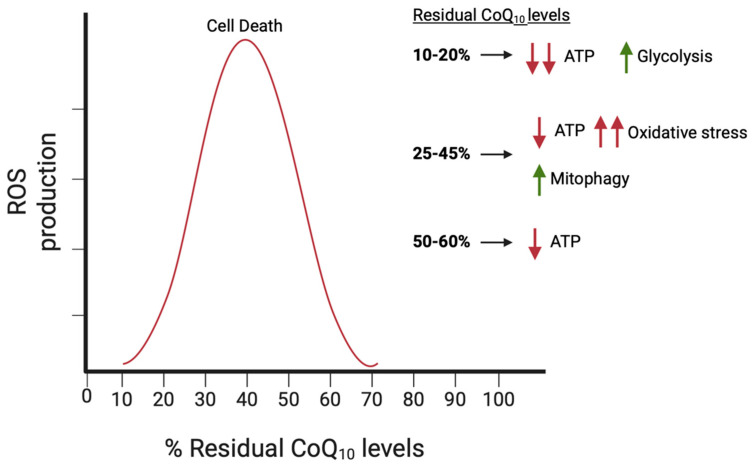
Schematic representation of ROS production as a function of residual CoQ_10_ concentration. Adapted from López et al. [218].

Because CoQ_10_ participates in both mitochondrial and non-mitochondrial antioxidant protection, its loss may contribute to oxidative damage affecting multiple cellular membranes. Indeed, a marked increase in ROS production has been demonstrated in human fibroblasts carrying mutations in various genes involved in CoQ_10_ biosynthesis. The group of Quinzii et al. has extensively investigated this issue and observed no correlation between the level of CoQ_10_ depletion and ROS generation [149,218,219,220,221]. They rather showed that ROS overproduction is most pronounced in moderate deficiencies of CoQ_10_ levels by approximately 40% (Figure 7).

The proposed explanation is that complete CoQ_10_ depletion markedly slows down the respiratory chain and shifts ATP production toward anaerobic glycolysis, whereas partial deficiencies allow for residual, yet dysfunctional, respiratory chain activity that produces higher levels of ROS. These findings, limited to fibroblast models, therefore indicate that ROS production is mainly secondary to respiratory chain dysfunction in the context of CoQ_10_ deficiency [149,219,220]. While a direct causal link between primary CoQ_10_ deficiency and ferroptosis has not yet been conclusively demonstrated, experimental disruption of CoQ_10_ transport from mitochondria to the plasma membrane was shown to markedly increase ferroptotic sensitivity [73]. In light of these findings on ROS production, it cannot be excluded that the oxidative stress and its associated damages would vary among cell types, which could explain why certain tissues are more susceptible to damage; this could explain why certain organs such as the brain, heart, or kidneys are more frequently affected in primary CoQ_10_ deficiencies. To further support this hypothesis, it would be of interest to investigate oxidative stress in tissues other than fibroblasts, using established markers of oxidative damage. These could include DNA oxidation products such as 8-oxo-dG, protein oxidation markers such as dityrosine, and indicators of lipid peroxidation such as isoprostanes.

In this context, the use of novel experimental models, such as iPSCs or organoid systems, could provide a particularly relevant approach. These models would allow the investigation of redox imbalance in a tissue-specific manner, better reflecting the cellular diversity of affected organs, and may help clarify the contribution of oxidative stress to the pathophysiology.

The link between primary CoQ_10_ deficiency and a reduction in these antioxidant functions has not been clearly demonstrated in humans. However, it is reasonable to assume that when the global CoQ_10_ pool decreases, not only are mitochondrial bioenergetics impaired, but also its antioxidant capacity may be compromised. In such a context, sustained oxidative stress, insufficiently counterbalanced by endogenous defense systems, could theoretically promote cell death pathways, including ferroptosis. This assumption is biologically consistent with the mechanisms previously discussed, which show that CoQH_2_ serves as a central lipid radical–trapping antioxidant, that its regeneration through enzymes such as FSP1 and DHODH constitutes a major ferroptosis-suppressive axis, and that mitochondrial complex I dysfunction can sensitize cells to ferroptosis by disturbing redox homeostasis and enhancing lipid peroxidation [193]. Thus, ferroptosis is likely to play a significant role in CoQ_10_ deficiencies. It may represent a major cell death pathway and could contribute to cellular damage, including cardiomyocyte necrosis [82] and neuronal loss associated with astrogliosis reported in *COQ4* mutations [76,83].

In conclusion, numerous studies link CoQ_10_ depletion to ROS levels, suggesting that oxidative stress is a major contributor to CoQ_10_ deficiency, as suggested for other mitochondrial diseases. In these cases, antioxidant therapies have yet to show clear clinical benefits [222]. It is important to note that these observations are limited to the specific molecules and outcome measures studied to date, such as visual improvement in Leber hereditary optic neuropathy. Thus, while oxidative stress is likely central to the disease’s pathophysiology, robust clinical evidence for the success of targeting this pathway remains elusive.

##### ROS as a Key Trigger of Mitophagy

Mitophagy is an essential cellular process that regulates mitochondrial mass through the autophagy pathway. It occurs under both physiological conditions, such as the elimination of mitochondria during erythropoiesis, and pathological situations, where it serves to remove damaged mitochondria. If not eliminated, these dysfunctional organelles can release pro-apoptotic signals, most notably cytochrome c, leading to cell death. The mitophagy process unfolds in several coordinated steps. First, dysfunctional mitochondria are recognized through specific molecular cues, often associated with fragmentation of the mitochondrial network. Once identified, they are isolated and subsequently enclosed within a forming autophagosome. The autophagosome then fuses with a lysosome, resulting in the degradation of mitochondrial components and the prevention of apoptosis [223]. Mitophagy plays a crucial role in maintaining mitochondrial quality control and has been implicated in a wide range of diseases, including Parkinson’s disease [224], cardiovascular disorders [225], and ischemic stroke [226]. Two major pathways for mitophagy activation have been described:

- The PINK1/Parkin pathway. This pathway is triggered by a loss of mitochondrial membrane potential. PINK1 and Parkin act in concert to label mitochondrial outer membrane proteins with phosphorylated ubiquitin. This ubiquitin tag is recognized by the autophagic machinery, allowing the recruitment of mitochondria to microtubule-associated protein 1A/1B-light chain 3 (LC3) through adaptor proteins and their subsequent incorporation into autophagosomes [227].
- The receptor-mediated pathways. Certain mitochondrial receptors, such as FUNDC1 under ischemic conditions, expose an LC3-interacting region (LIR) motif upon activation. This motif directly binds LC3, forming a bridge between the mitochondrion and the autophagosome [228]. These receptors function in a context-dependent manner, responding to specific types of cellular stress or damage.

Few studies have investigated the relationship between CoQ_10_ deficiency and mitophagy. Nevertheless, this connection is biologically plausible, as several pathophysiological mechanisms observed in CoQ_10_ deficiency could also activate mitophagy. These include mitochondrial membrane depolarization, which may trigger the PINK1/Parkin pathway, and excessive oxidative stress leading to mitochondrial damage and mitophagy induction.

The first study to explore this relationship demonstrated a disruption of the mitochondrial network, a preliminary step required for the selective targeting of damaged mitochondria [149,229]. The authors analyzed fibroblasts from four patients carrying *COQ* gene mutations and observed decreased mitochondrial membrane potential (less polarized mitochondria), a key signal for mitophagy activation. Increased ROS production was also noted, further contributing to mitochondrial injury and mitophagy initiation. Consistent with this, expression of autophagy-related proteins (LC3, Beclin, and Atg12) was upregulated, lysosomal activity was enhanced, as evidenced by increased acidic vacuoles and lysotracker staining, and electron microscopy revealed autophagosomes containing mitochondria. Importantly, treatment with the antioxidant N-acetylcysteine (NAC) attenuated autophagy, confirming the role of oxidative stress as a trigger [229].

This study was the first to implicate mitophagy in the context of primary CoQ_10_ deficiency. The functional role of mitophagy in this setting remains to be fully defined. As demonstrated in other pathologies, mitophagy generally exerts a cytoprotective function by eliminating damaged mitochondria and preventing the release of pro-apoptotic factors. However, excessive or sustained activation may lead to mitochondrial depletion and impaired bioenergetic capacity, potentially worsening cellular dysfunction. Further studies focusing on the specific actors of mitophagy, particularly PINK1 and Parkin, are required to better characterize the mechanisms involved in CoQ_10_ deficiency. However, it has been demonstrated in certain mitochondrial diseases, such as Leber’s hereditary optic neuropathy, that mitophagy can become persistently activated and likely contributes to a profound depletion of mitochondrial mass, as it is not adequately compensated by mitobiogenesis, ultimately leading to neuronal degeneration. Mitophagy is therefore not inherently protective and may exacerbate the pathological condition when excessively sustained. Notably, interventions aimed at reducing mitophagy, such as supplementation with CoQ_10_ precursors to mitigate oxidative stress, have been shown to exert a beneficial effect on cellular survival. Together, these findings show that while mitophagy serves a protective function, its excessive activation can promote mitochondrial depletion and contribute to cell death [230].

Because this cellular mechanism is involved in the removal of damaged mitochondria, it is expected to be activated in the context of CoQ_10_ deficiency. While mitophagy is initially protective, its sustained or excessive activation may become deleterious, as it can lead to excessive mitochondrial depletion and consequently to cellular energy deficiency. The most important trigger for mitophagy activation appears to be the accumulation of ROS, suggesting that mitophagy represents a secondary mechanism downstream of oxidative stress. Based on the current body of evidence, this therefore supports the view that mitophagy constitutes a minor pathophysiological mechanism in CoQ_10_ deficiency rather than a primary driver of disease. However, the dynamics of mitophagy in CoQ_10_ deficiency remain insufficiently characterized. Many of the tools used in published studies rely on general autophagy markers, while relatively few directly assess the core components of the mitophagy machinery, such as PINK1 and Parkin. Further investigations are therefore required to better understand the role of mitophagy in primary CoQ_10_ deficiencies.

#### 5.2.4. Role of Inflammation in Coenzyme Q_10_ Deficiency

Inflammation is a physiological process that contributes to host defense and the restoration of homeostasis in response to various insults. This tightly regulated system is initiated by specific sensing mechanisms, particularly in the context of tissue stress, where damage-associated molecular patterns (DAMPs), including those of mitochondrial origin, play a central role [231]. The onset of inflammation involves several classes of pattern recognition receptors (PRRs). These receptors detect pathogen-associated molecular patterns (PAMPs) and DAMPs released from stressed or damaged cells. Among extracellular PRRs, Toll-like receptors (TLRs) and C-type lectin receptors (CLRs) are key sensors. Intracellularly, other PRRs such as RIG-I-like receptors (RLRs), AIM2-like receptors (ALRs) and NOD-like receptors (NLRs) contribute to the detection of nucleic acids, lipids and other danger signals. Activation of membrane or endosomal PRRs typically engages the NF-κB pathway and induces the transcription of pro-inflammatory cytokines. Moreover, activation of cytosolic NLRs can lead to the assembly of inflammasomes, which activate caspase-1 and promote the maturation and secretion of IL-1β and IL-18, often associated with lytic forms of cell death such as pyroptosis [232].

Mitochondrial stress or damage can lead to the release of mtDNA into the cytosol or extracellular environment. Once outside the mitochondrial matrix, mtDNA behaves as a DAMP that is sensed by innate immune receptors. Cytosolic mtDNA may activate the cGAS–STING signaling pathway, triggering production of type I interferons and pro-inflammatory cytokines. In parallel, extracellular or endosomal mtDNA can engage receptors such as TLR9 or stimulate inflammasome complexes (e.g., NLRP3), leading to secretion of IL-1β and IL-18 and amplification of the inflammatory response. Thus, mitochondrial dysfunction, through mtDNA release, constitutes a mechanistic bridge between mitochondrial damage and sterile inflammation [233].

Indirect evidence suggests a link between CoQ_10_ and inflammation. Low CoQ_10_ levels are associated with NLRP3 activation in peripheral blood mononuclear cells obtained from patients affected by fibromyalgia, and supplementation by exogenous CoQ_10_ reverses these abnormalities [234]. In preclinical models, CoQ_10_ and its analogs (CoQ_0_) inhibit NLRP3 activation by reducing mitochondrial ROS, suppressing NF-κB signaling, and, in some cases, promoting autophagy [235,236]. Notably, interventions aimed at reducing autophagy reversed CoQ_0_-induced inhibition of inflammation. Together, these findings showed that in such a context, autophagy had a positive effect.

To date, no study has specifically and systematically characterized the inflammatory response in patients with primary CoQ_10_ deficiency. However, several mechanistic hypotheses can be proposed based on the known pathophysiology of CoQ_10_ deficiency and on general links between mitochondrial dysfunction and inflammation:

- ROS-mediated activation of inflammasome: increased ROS production, observed in primary CoQ_10_ deficiency, is a well-established trigger of inflammasome activation. In particular, ROS can promote dissociation of the thioredoxin (Trx)–thioredoxin-interacting protein (TXNIP) complex, allowing TXNIP to bind NLRP3 and drive NLRP3 inflammasome assembly, caspase-1 activation and IL-1β/IL-18 release [237].
- Role of Mitochondrial DAMPs in inflammasome activation: several mitochondrial components have been shown to trigger inflammatory responses through activation of the inflammasome [231]. Although a direct link between ferroptosis and the release of these mitochondrial danger signals has not yet been demonstrated, this remains a plausible scenario given the profound disruption of mitochondrial membranes during lipid peroxidation and the potential extrusion of pro-inflammatory factors such as oxidized cardiolipin [238].
- Altered sulfide oxidation and H_2_S accumulation: H_2_S has been identified as a mediator of inflammation, and several studies have reported a correlation between elevated H_2_S levels and the severity of inflammatory responses [239]. Therefore, in primary CoQ_10_ deficiency, where disturbances in H_2_S metabolism have been observed, it is plausible that altered H_2_S signaling represents a contributing mechanism to the associated inflammatory phenotype.

In conclusion, multiple lines converge toward a probable overactivation of inflammatory pathways in primary CoQ_10_ deficiencies, potentially driven by ROS-induced cellular damage, the release of DAMPs, and the disturbance in sulfide metabolism. However, the mechanism still requires further validation through additional experimental investigation, and its clinical relevance remains to be established through appropriately designed therapeutic studies. Based on the current evidence, we can therefore classify this pathophysiological mechanism as minor.

### 5.3. Physiopathological Hypothesis About Tissue Susceptibility

**Figure 8:**
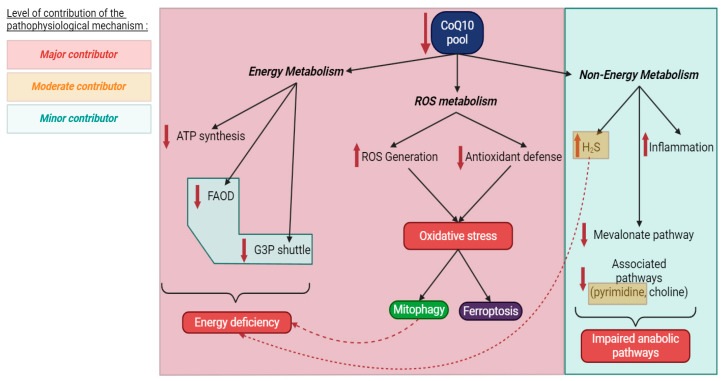
Main hypotheses and metabolic pathways that may explain the pathophysiological mechanisms of primary coenzyme Q_10_ deficiencies. The pathophysiological mechanisms are shown on a colored background according to their relative importance in the pathophysiology of primary CoQ_10_ deficiency. FAOD: Fatty acid oxidation disorder. Dotted arrows mean indirect pathophysiological mechanisms. Created in BioRender. https://BioRender.com/, accessed on 20 December 2025.

Primary CoQ_10_ deficiencies illustrate how disruption of a single metabolic hub can trigger a cascade of interconnected cellular dysfunctions. Although CoQ_10_ depletion is biochemically defined by impaired electron transfer within the MRC, the clinical and experimental evidence reviewed here clearly demonstrates that disease pathophysiology extends far beyond an isolated defect in ATP production. Conversely, CoQ_10_ deficiency triggers multiple interconnected pathways (Figure 8). Their varying contributions to the pathophysiology of these diseases collectively dictate tissue vulnerability, disease severity, and phenotypic expression.

#### 5.3.1. Differential Depletion of CoQ_10_ Among Tissues

While human data are limited due to the invasive nature of organ sampling, preclinical models with human *COQ* gene mutations demonstrate that CoQ_10_ depletion levels are both organ- and mutation-specific [240]. For instance, in Coq9^Q95X^ mice, depletion is more severe in skeletal muscle than in the brain or cerebellum. Comparing the Coq9^Q95X^ and Coq9^R239X^ models reveals that the latter induces greater depletion across the brain, heart, liver, and kidneys, though not in skeletal muscle.

**Figure 9:**
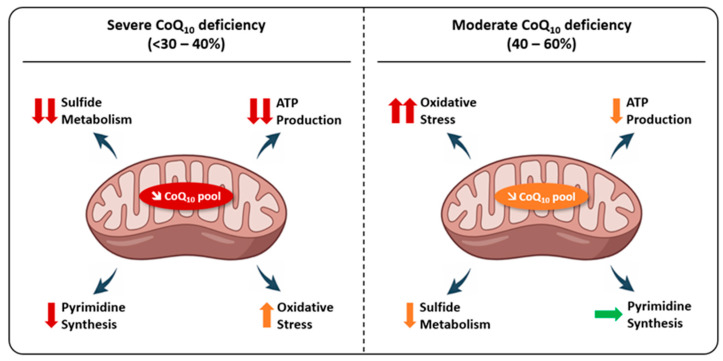
Threshold-dependent effects of CoQ_10_ deficiency on mitochondrial metabolism and tissue vulnerability. Severe CoQ_10_ deficiency (<30–40% of normal) leads to a major decrease in ATP production, profound impairment of sulfide oxidation due to SQOR instability, and reduced DHODH-dependent pyrimidine synthesis, while ROS production remains moderately elevated. By contrast, moderate CoQ_10_ deficiency (≈40–60% of normal) causes a moderate decrease in ATP production, partial impairment of sulfide oxidation, and marked increases in ROS generation. Pyrimidine synthesis is largely preserved in this context. Arrow thickness represents the relative magnitude of pathway alterations, and color coding indicates the severity of dysfunction (red, severe; orange, moderate; green, preserved). Created in BioRender. https://BioRender.com/, accessed on 20 December 2025.

These distinct depletion profiles dictate the pathological outcome: partial deficiency primarily causes tissue damage through oxidative stress, due to the loss of radical scavenging and impaired antioxidant regeneration, rather than through primary energy failure (Figure 9). Elevated oxidative stress damages mitochondrial lipids, proteins, and DNA, further impairing mitochondrial function and creating a self-reinforcing loop of dysfunction. Persistent redox imbalance can activate apoptotic and necrotic cell death pathways, contributing to progressive tissue degeneration, particularly in post-mitotic tissues such as neurons and cardiomyocytes. In the case of a severe CoQ_10_ depletion leading primarily to ATP deficiency (Figure 9), numerous cellular dysfunctions occur, including a drastic reduction in muscle contraction, particularly cardiac contraction; the shutdown of ATP-dependent ion pumps both on the cell surface and in intracellular compartments, leading to a massive accumulation of calcium in the cytosol and mitochondria; and an accumulation of sodium and water inside the cell, leading to cell swelling and cell death by necrosis. The release of intracellular content into the extracellular environment will constitute an additional signal, thus creating inflammation in neighboring cells.

#### 5.3.2. Energy Requirement

The most direct consequence of CoQ_10_ depletion is impaired electron transport between several mitochondrial dehydrogenases and complex III, leading to reduced respiratory chain efficiency and ATP synthesis. The severity of this energetic defect depends on residual CoQ_10_ levels (Figure 9) and on the degree to which tissues rely on oxidative phosphorylation. Experimental data from patient-derived cells show that mild to moderate CoQ_10_ depletion primarily limits maximal respiratory capacity, whereas severe deficiency compromises basal respiration, mitochondrial membrane potential, and ATP/ADP ratios, particularly under conditions that force reliance on oxidative metabolism. These findings would explain why organs with high and continuous energy demands, such as the brain, heart, skeletal muscle, and kidney, are preferentially affected. However, the existence of isolated organ phenotypes indicates that energy failure alone is insufficient to account for the full clinical spectrum.

#### 5.3.3. Different Levels of Antioxidant Enzymes

Tissue susceptibility to oxidative stress is determined by the interplay of several factors: the expression of antioxidant enzymes or molecules, including CoQ_10_ concentrations; the prevalence of damage-prone structures like lipid-rich membranes; and the intrinsic rate of ROS production, which is closely linked to mitochondrial density and metabolic demand.

Within the superoxide dismutase family, SOD1 (cytosolic) and SOD2 (mitochondrial) are ubiquitously expressed. In contrast, SOD3 (extracellular) shows a more restricted distribution, peaking in vascular tissues, notably at the blood–brain barrier, where it likely maintains extracellular redox homeostasis [241]. Regarding systems more closely tied to CoQ_10_ metabolism, GPX4 is notably enriched in lipid-heavy organs such as the brain, liver, and heart, though exact quantitative expression is still being defined [205]. Conversely, FSP1 (AIFM2) is generally expressed at low levels, with only a slight predominance in the liver. Whether this profile indicates a true tissue-specific reliance on the FSP1–CoQ_10_ axis remains a subject for further study.

The brain serves as a primary example of high oxidative vulnerability. This sensitivity stems from its dense lipid composition, high energetic requirements, and heavy reliance on mitochondrial metabolism, all of which drive elevated ROS production. Furthermore, evidence suggests that the brain’s antioxidant defenses may be relatively sparse compared to other metabolically active tissues [242].

Collectively, these observations suggest that tissue-specific redox architecture may dictate differential vulnerability to ROS. This variation represents a significant, yet underexplored, factor in explaining the clinical heterogeneity of CoQ_10_ deficiency. However, direct evidence linking tissue-specific ROS susceptibility to clinical phenotypes remains sparse, necessitating further experimental validation.

#### 5.3.4. Differential Pathways

As previously discussed, CoQ_10_ occupies a central position at the intersection of multiple metabolic pathways, meaning its depletion can trigger diverse cellular alterations depending on the specific requirements of the affected tissue. In energy-demanding cardiac and skeletal muscles, CoQ_10_ deficiency impairs electron transfer from ETFDH, thereby reducing mitochondrial β-oxidation. This metabolic block leads to lipid accumulation, myopathy, and cardiomyopathy, as documented in both clinical and preclinical models.

Furthermore, studies in iPSC-derived neurons indicate that CoQ_10_ loss disrupts the mevalonate pathway and cholesterol trafficking. Such perturbations in lipid homeostasis likely compromise membrane composition, which is particularly detrimental to neurons given their reliance on membrane dynamics for myelination and synaptic function.

Additionally, the tissue-specific expression of CoQ_10_-dependent enzymes explains varying organ susceptibility. For instance, SQOR is highly expressed in the kidneys and skeletal muscle. Under conditions of CoQ_10_ deficiency, SQOR is destabilized, which impairs mitochondrial hydrogen sulfide detoxification. The resulting accumulation of sulfide and sulfane sulfur species further inhibits respiratory complexes and β-oxidation while altering redox signaling. Much like ATP synthesis and ROS production, this pathway is highly sensitive to CoQ_10_ levels, potentially acting as a critical amplifier of mitochondrial dysfunction once a specific depletion threshold is reached.

#### 5.3.5. Exogenous Intake of CoQ_10_

Another factor that could partly explain the differential damage to certain tissues compared to others is the contribution of exogenous CoQ_10_. While CoQ_10_ supplementation (200 mg) for 14 days in healthy human subjects does not increase the muscle concentration of this cofactor [243], oral supplementation (between 20 and 73 mg/kg/day) does increase CoQ_10_ concentrations measured in PBMCs from patients with primary deficiency [244]. It can therefore be assumed that, in physiological conditions, the contribution of exogenous CoQ_10_ to tissue concentrations is negligible, but that in pathological conditions (i.e., primary CoQ_10_ deficiency), this pathway would allow for an intake, albeit insufficient to enable subnormal cellular function. Given that exogenous CoQ_10_ is transported mainly by lipoproteins in the blood, HDL, LDL, and CD36 receptors could be involved in the tissue import of CoQ_10_, as suggested by preclinical studies [245]. However, this hypothesis has not been validated by the work of Saiki and colleagues on the Pdss2^kd/kd^ mouse model. Although a reduction in proteinuria is observed after CoQ_10_ supplementation, no increase in this cofactor has been observed in the kidney [246].

### 5.4. Translational Implications

Primary CoQ_10_ deficiency is unique among mitochondrial diseases, as it is the only one considered treatable through cofactor supplementation. Theoretically, administering exogenous CoQ_10_ should bypass the metabolic block caused by pathogenic variants in *COQ* genes, restoring tissue levels and the biochemical reactions dependent on them. Indeed, the capacity to rescue these metabolic pathways is a key criterion for confirming the pathogenicity of newly identified variants.

Clinical outcomes, however, vary significantly. In a cohort of patients with ataxia caused by *COQ8A* mutations, Traschütz et al. found that approximately 50% responded positively to daily CoQ_10_ doses (2–40 mg/kg/day) with minimal side effects [97]. Interestingly, the response was independent of patient age, age of onset, or disease duration. Clinical improvements were confirmed through longitudinal assessments, where symptoms often worsened if treatment was discontinued. Recent systematic reviews also highlight the complexities of CoQ_10_ treatment efficacy. Wang and Hekimi analyzed 89 well-documented cases and found a responder rate of only 27% [247]. This low percentage may result from the difficulty of evaluating a positive response in patients affected by multi-systemic symptoms. However, they noted that the response was independent of dose or duration, with isolated renal forms and less severe phenotypes showing the best outcomes. In a critical review of over 330 patients harboring various *COQ* mutations, Mantle et al. highlighted the necessity of early CoQ_10_ supplementation to prevent irreversible tissue damage [248]. They found that therapeutic efficacy is highly gene-specific, showing significant success in COQ6-related nephropathy, but remains limited in treating CNS symptoms, likely due to the poor permeability of CoQ_10_ across the blood–brain barrier.

Mechanistically, cellular models suggest that the “dose–response” relationship is not uniform across all biological functions. Duberley et al. demonstrated that while low concentrations of CoQ_10_ can reduce superoxide production, significantly higher doses are required to restore mitochondrial membrane potential or to partially correct respiratory chain deficiencies [249]. This suggests that the clinical success of supplementation depends heavily on whether the exogenous cofactor can reach specific tissues and penetrate the relevant cellular compartments, such as the mitochondria.

These data underscore the necessity of early intervention and the use of objective criteria to monitor treatment efficacy in suspected primary CoQ_10_ deficiency. Future research has to focus on enhancing the bioavailability of CoQ_10_ and elucidating its transport mechanisms to different organs and intracellular spaces to more effectively target the pathophysiology of these deficiencies.

## 6. Conclusions

Ultimately, primary CoQ_10_ deficiencies transcend simple mitochondrial energy failure; they are multisystemic conditions driven by complex metabolic and redox imbalances. Clinical manifestations appear to be governed by tissue-specific vulnerability, likely dictated by varying degrees of residual CoQ_10_ and dysfunctions in cellular pathways. Nevertheless, certain questions remain unanswered and open up essential avenues of research for improving more effective and targeted therapeutic management of patients with primary deficiency.

### 6.1. Does the Efficacy of CoQ_10_ Supplementation Vary Across Different Organs?

Studies using ^11^C-labeled CoQ_10_ demonstrate preferential accumulation in the liver and heart, with significantly lower uptake in skeletal muscle and the brain. These distribution patterns suggest that while oral supplementation may effectively address cardiac or renal manifestations of primary CoQ_10_ deficiency, its efficacy for neurological symptoms remains limited. Consequently, future efforts must focus on enhancing CoQ_10_’s blood–brain barrier permeability or engineering therapies that target tissue-specific cellular dysfunction directly.

### 6.2. Does CoQ_10_ Supplementation in Patients with Primary Deficiency Correct Both Mitochondrial and Extramitochondrial Concentrations?

While CoQ_10_ is primarily localized within the mitochondrial fraction, it is also present in the non-mitochondrial pool, where it primarily serves as an antioxidant and ferroptosis inhibitor. No study has yet determined whether CoQ_10_ depletion in patients with biosynthesis defects affects the mitochondrial pool, the non-mitochondrial pool, or both. Furthermore, it remains to be determined whether exogenous CoQ_10_ supplementation can effectively or preferentially restore these specific cellular pools.

### 6.3. Is It Possible to Measure the Involvement of the Various Pathways or Reactions in Which CoQ_10_ Is Involved in Pathogenicity in Humans?

As previously noted, CoQ_10_ is involved in several biochemical pathways, such as pyrimidine synthesis and choline metabolism, whose roles in specific pathophysiological mechanisms remain unknown due to a lack of objective data. We propose that organoids derived from patient-specific iPSCs represent a promising experimental model to address this gap. Using these models, specialized biochemical assays could be developed to measure pathway-specific metabolites with greater precision. Furthermore, pharmacological interventions, specific gene expression or CRISPR-Cas9 gene editing could be employed to correct observed alterations, allowing for the evaluation of therapeutic outcomes at both the cellular and organoid levels.

### 6.4. Is There a Tissue-Specific Critical Threshold of CoQ_10_?

It is well admitted that the degree of CoQ_10_ depletion, which varies across different tissues, directly dictates cellular dysfunction and, consequently, clinical phenotype (as illustrated in Figure 8 and Figure 9). A critical consideration is that pathological thresholds for CoQ_10_ may be tissue-specific; investigating these distinct thresholds could provide an interesting insight into the underlying pathophysiological mechanisms of the disease.

### 6.5. Given That the Pathophysiological Mechanisms of Primary CoQ_10_ Deficiencies Are Complex and Involve Several Pathways, Would Combination Therapies Targeting These Different Pathways Be Effective?

Clinical outcomes for patients with mutations in CoQ_10_ biosynthesis genes remain inconsistent, as supplementation often fails to yield uniform benefits. This suggests that instead of focusing exclusively on restoring CoQ_10_ levels, therapeutic efforts should also expand to address secondary cellular disruptions, including neuroinflammation, oxidative stress, H_2_S metabolism, and cell death signaling. Integrating CoQ_10_ with specific modulators of these pathways may provide a more robust and effective therapeutic framework than monotherapy with the cofactor alone.

## Data Availability

No new data were created or analyzed in this study.

## Abbreviations

The following abbreviations are used in this manuscript:

8-oxo-dG	8-oxo-2′-deoxyguanosine
4-HBF	4-hydroxybenzoylformate
4-HB	4-hydroxybenzoate
4-HBz	4-hydroxybenzaldehyde
4-HMA	4-hydroxymandelate
4-HNE	4-hydroxynonenal
4-HPP	4-hydroxyphenylpyruvate
AADAT	α-aminoadipate aminotransferase
ADCK	AarF domain-containing kinase
ADP	Adenosine diphosphate
ALDH3A1	Aldehyde dehydrogenase 3 family member A1
ALRs	AIM2-like receptors
ATP	Adenosine triphosphate
ATPase	adenosine triphosphatase
BADH	Betaine aldehyde dehydrogenase
BHMT	Betaine-homocysteine methyltransferase
CDP-choline	Cytidine diphosphate choline
CHDH	Choline dehydrogenase
CI	Mitochondrial respiratory chain complex I
CIV	Mitochondrial respiratory chain complex IV
CLRs	C-type lectin receptors
CNS	Central nervous system
CoQ	Coenzyme Q
CoQH_2_	Ubiquinol
COX	Cytochrome c oxidase
Cryo-EM	Cryo-electron microscopy
CTP	Cytidine triphosphate
CYB5R	Cytochrome b5 reductase
DAMPs	Damage-associated molecular patterns
DDMQ	Demethoxy-demethyl-CoQ_10_
DHAP	Dihydroxyacetone phosphate
DHODH	Dihydroorotate dehydrogenase
DMeQ	Demethyl-CoQ_10_
DMAP	Dimethylallyl pyrophosphate
DMQ	Demethoxy-CoQ_10_
DN	Deoxyribonucleic acid
D2HGDH	D-2-hydroxyglutarate dehydrogenase
ETC	Electron transport chain
ETF	Electron-transfer flavoprotein
ETFDH	Electron-transferring flavoprotein dehydrogenase
ETF-QO	Electron-transfer flavoprotein–ubiquinone oxidoreductase
ETHE1	Ethylmalonic encephalopathy protein 1
E–SSH	Protein persulfide
FAD	Flavin adenine dinucleotide
FADH_2_	Reduced flavin adenine dinucleotide
FBS	Fetal bovine serum
FMN	Flavin mononucleotide
FMNH_2_	Reduced flavin mononucleotide
FPPS	Farnesyl diphosphate synthase
FPP	Farnesyl pyrophosphate
FSP1	Ferroptosis suppressor protein 1
FUNDC1	FUN14 domain-containing protein 1
G3P	Glycerol-3-phosphate
GPX	Glutathione peroxidase
GPD1	Glycerol-3-phosphate dehydrogenase 1
GPD2	Glycerol-3-phosphate dehydrogenase 2
GPP	Geranyl pyrophosphate
GR	Glutathione reductase
GSH	Reduced glutathione
GSSH	Glutathione persulfide
GSTs	Glutathione-S-transferases
HDL	High-density lipoprotein
HEK293	Human embryonic kidney 293
HMGCR	3-hydroxy-3-methylglutaryl-CoA reductase
HPDL	Hydroxyphenylpyruvate dioxygenase-like protein
IMS	Intermembrane space
IMM	Inner mitochondrial membrane
IPP	Isopentenyl pyrophosphate
iPSC	Induced pluripotent stem cells
IUGR	Intrauterine growth restriction
LC3	Microtubule-associated protein 1 light chain 3
LDHD	Lactate dehydrogenase D
LDL	Low-density lipoprotein
LIR	LC3-interacting region
MADD	Multiple acyl-CoA dehydrogenase deficiency
MDA	Malondialdehyde
MRC	Mitochondrial respiratory chain
mtDNA	Mitochondrial DNA
NAC	N-acetylcysteine
NAD^+^	Nicotinamide adenine dinucleotide
NADH	Reduced nicotinamide adenine dinucleotide
NADP	Nicotinamide adenine dinucleotide phosphate
NF-κB	Nuclear factor kappa-B
NLRP3	NLR family pyrin domain-containing 3
NLRs	NOD-like receptors
NQO1	NAD(P)H quinone dehydrogenase 1
OMM	Outer mitochondrial membrane
PAH	Phenylalanine hydroxylase
PAMPs	Pathogen-associated molecular patterns
PBHQ	3-polyprenyl-5-hydroxy-1,4-benzohydroquinone
PBQ	3-polyprenyl-1,4-benzohydroquinone
PDSS1	Decaprenyl diphosphate synthase subunit 1
PDSS2	Decaprenyl diphosphate synthase subunit 2
PINK1	PTEN-induced kinase 1
PMRS	Plasma membrane redox system
PPDHB	3-polyprenyl-4,5-dihydroxybenzoate
PPHB	3-polyprenyl-4-hydroxybenzoate
PPMP	2-polyprenyl-6-methoxyphenol
PPP	Polyprenyl pyrophosphate
PPVA	3-polyprenylvanillic acid
PRRs	Pattern-recognition receptors
PRODH1	Proline dehydrogenase 1
PRODH2	Proline dehydrogenase 2
PUFAs	Polyunsaturated fatty acids
Q	Ubiquinone
QH2	Ubiquinol
RET	Reverse electron transport
RIG-I	Retinoic acid-inducible gene I
RLRs	RIG-I-like receptors
RNA	Ribonucleic acid
ROS	Reactive oxygen species
SAM	S-adenosylmethionine
SCAD	Short-chain acyl-CoA dehydrogenase
SCs	Respiratory supercomplexes
SOD1	Superoxide dismutase 1
SOD2	Superoxide dismutase 2
SQ	Semiquinone
SQOR	Sulfide quinone oxidoreductase
SUOX	Sulfite oxidase
TAT	Tyrosine aminotransferase
TCA	Tricarboxylic acid
TLR9	Toll-like receptor 9
TLRs	Toll-like receptors
Trx	Thioredoxin
TST	Thiosulfate sulfurtransferase
TXNIP	Thioredoxin-interacting protein
UMP	Uridine monophosphate
UTP	Uridine triphosphate
VLDL	Very-low-density lipoprotein
